# Synergistic effects of a sequential recirculation electrochemical system combined with low-cost UV-LEDs on the gram-negative bacteria inactivation

**DOI:** 10.1007/s11356-024-35297-0

**Published:** 2024-12-21

**Authors:** Paula Andrea Espinosa-Barrera, Efraím A. Serna-Galvis, Ricardo Antonio Torres-Palma, David Izquierdo-Sandoval, Félix Hernández, Diana Martínez-Pachón, Alejandro Moncayo-Lasso

**Affiliations:** 1 Grupo de Investigación en Ciencias Biológicas y Químicas, Facultad de Ciencias, Universidad Antonio Nariño, Bogotá D.C, Colombia; 2 Doctorado en Ciencia Aplicada - DCA, Universidad Antonio Nariño, Bogotá D.C, Colombia; 3 Grupo de Investigación en Remediación Ambiental y Biocatálisis (GIRAB), Instituto de Química, Facultad de Ciencias Exactas y Naturales, Universidad de Antioquia UdeA, Calle 70 No. 52-21, Medellín, Colombia; 4 Grupo de Catalizadores y Adsorbentes (CATALAD), Instituto de Química, Facultad de Ciencias Exactas y Naturales, Universidad de Antioquia UdeA, Calle 70 No. 52-21, Medellín, Colombia; 5 Environmental and Public Health Analytical Chemistry, Research Institute for Pesticides and Water, University Jaume I, Castellón, Spain

**Keywords:** Electrochemical process, Flow cytometry, Gram-negative bacteria, LEDs, Membrane disruption, Synergistic combination

## Abstract

In this work, an electrochemical system combined with low-cost UV-LEDs was implemented for the inactivation of *Escherichia coli* and *Pseudomonas aeruginosa*. The individual elimination of these bacteria was followed by plate counting and flow cytometry, as complementary techniques to establish the cell inactivation and non-viability, respectively. The contribution of the different parts of the disinfection system (anode, cathode, and light) was determined. In addition, the efficiency of the UV-LEDs/GDE/DSA system in the disinfection of an irrigation water sample was studied. It was found that the combination of the electrochemical system with UV-LEDs was highly synergistic (φ > 7), having low electric energy consumptions per order of magnitude (E_EO_: 1.13 × 10^–2^ and 1.55 × 10^–2^ kWh/m^3^ order). Moreover, some differences in the inactivation kinetics and synergy between *E. coli* and *P. aeruginosa* were observed and linked to the structural/morphological characteristics of the two bacteria. Remarkably, the electrochemical system combined with low-cost UV-LEDs inactivated both target microorganisms after only 2 min of treatment. The flow cytometry analyses evidenced the damage to the cell membrane of the bacteria by the simultaneous and synergistic action of the electrogenerated H_2_O_2_ and active chlorine species (ACS), plus the attacks of photo-generated reactive oxygen species. This synergistic combination in the UV-LEDs/GDE/DSA system demonstrated remarkable efficiency in the disinfection of an irrigation water sample, achieving the elimination of culturable bacteria in 45 min of treatment. The results of this research demonstrated the capacity and great potential of an easy combination of electrochemistry with UV-LEDs as an alternative system for the elimination of gram-negative bacteria in water.

**Supplementary Information:**

The online version contains supplementary material available at 10.1007/s11356-024-35297-0.

## Introduction

In recent years, rapid urbanization, population expansion, and the intensification of human activities have resulted in significant burdens on global water resources, leading to a deterioration of water quality through chemical (presence of contaminants of emerging concern) and microbiological contamination. This problem is relevant even in countries with high sanitation standards (Chen et al. 2021; Delgado-Vargas et al. 2023).

The presence of contaminants of emerging concern (CEC) and an increase in waterborne diseases (WBD), including foodborne diseases (FBD) when crops have been irrigated with contaminated water, which even has been previously treated with conventional processes. Several parameters of water quality are usually related to microbial contamination, primarily due to the presence of pathogens such as *Escherichia coli* and *Pseudomonas aeruginosa*. These two gram-negative bacteria are model microorganisms, as they are among the most abundant pathogens present in different wastewaters around the world. Such bacteria have been detected in several WBD outbreaks, and can easily develop resistance to multiple antibiotics (Fakhkhari et al. 2022; Numberger et al. 2019).

The ubiquity of pathogens and CEC in water bodies requires the use of technologies that ensure their elimination and inactivation, thus improving sanitation and public health (López et al. 2017). Tertiary treatments are often required because conventional treatments do not efficiently eliminate these contaminants. Some conventional water treatments include disinfection methods, such as hydrogen peroxide (H_2_O_2_), chlorination with chlorine gas (Cl_2(g)_), or salts derived from hypochlorous acid (HClO), as well as ultraviolet radiation (UV), which are widely applied in wastewater treatment facilities either separately or in binary combination (e.g., UV/H_2_O_2_ and UV/Cl_2_). These methods have demonstrated high efficiency in inactivating a wide spectrum of microorganisms, including bacteria, fungi, viruses, and bacteriophages. In addition, they can also eliminate genes associated with pathogenicity, such as those responsible for DNA repair, resistance to antibiotics, and production and secretion of toxins, among others (Ferro et al. 2016; Zeng et al. 2020). However, implementing such disinfection methods presents several technical challenges. These include the continuous addition of reagents (e.g., H_2_O_2_, Cl_2(g),_ and/or HClO salts), which can increase operational expenses, along with high energy consumption when implementing UV mercury lamps, due to their short useful life and high purchasing costs. Furthermore, disposing of damaged UV lamps is also problematic (Chen et al. 2021) and does not guarantee CEC removal.

To overcome some of these technical challenges of the above-mentioned disinfection systems, and promote the removal of CEC, an electrochemical process can be utilized. This process enables the continuous in situ generation of oxidizing species. These species can include H_2_O_2_ (E° = 1.77 vs SHE), and active chlorine species (ACS), such as HClO (E° = 1.49 V vs SHE) and hypochlorite anion (ClO^−^, E° = 0.89 V vs SHE) (Antonelli et al. 2022; Comninellis and Chen 2010; Cordeiro-Junior et al. 2022; Palma-Goyes et al. 2022). H_2_O_2_ is electrogenerated on the surface of a gas diffusion cathode (GDE) through oxygen reduction (Eq. 1) (Bavasso et al. 2020; Cordeiro-Junior et al. 2022). Meanwhile, ACS can be electrogenerated at a dimensionally stable anode (DSA) surface, specifically during the electrolysis of aqueous chloride solutions. Initially, there is direct oxidation of chloride ion (Cl^−^) at the anode surface, resulting in the generation of Cl_2(aq)_ (Eq. 2). Subsequently, Cl_2(aq)_ is rapidly disproportionated, in the aqueous medium, into HClO and HCl (Eq. 3). Additionally, HClO can form an acid/base equilibrium with ClO^−^ (Eq. 4) (Ayadi et al. 2023; Ferreira de Melo et al. 2020; Murrieta et al. 2023; Scialdone et al. 2021).

1 $${\text{O}}_{2} + {2\text{H}}^{+} + {2\text{e}}^{-}\to {\text{H}}_{2}{\text{O}}_{2}$$

2 $${2\text{Cl}}^{-} \to {\text{Cl}}_{2(\text{aq})} + {2\text{e}}^{-}$$

3 $${\text{Cl}}_{2(\text{aq})}+ {\text{H}}_{2}\text{O}\to \text{HClO }+\text{ HCl}$$

4 $$\text{HClO }+{\text{H}}_{2}\text{O}\leftrightarrow \text{ClO}+{{\text{H}}_{3}\text{O}}^{+}$$

The ACS speciation depends on the pH and two important equilibria are related to the transitions Cl_2_/HClO (pK_a_ = 2.2) and HClO/ClO^−^ (pK_a_ = 7.54). Under acidic conditions or high applied currents, the production of Cl_2(g)_ can be favored, which is a competitive reaction to produce ACS. To avoid these parasitic reactions, the use of an electrochemical system at neutral or circumneutral pH is recommended, promoting the highest concentration of HClO, which is a strong oxidant (Ferreira de Melo et al. 2020). This represents the minimum viable requirement, i.e., the essential conditions for the system to operate with high efficiency in water disinfection.

On the other hand, the implementation of UV-emitting LEDs addresses some of the issues associated with the use of UV mercury lamps. LEDs offer lower energy consumption and lower acquisition costs, have a longer lifespan, and are recyclable at the end of their life. Therefore, UV-LEDs-assisted electrochemical systems can be a combined oxidation process for the elimination of microorganisms in water samples (Chaplin 2018; Coha et al. 2021).

Several systems that use UVC- or UVB-LEDs alone or in combination with H_2_O_2_ or ACS are recognized as efficient treatments for bacteria inactivation in water (Li et al. 2019; Moreno-Andrés et al. 2023; Wu et al. 2021). Also, some previous studies have reported the utilization of photo-electrochemical systems employing UV light based on mercury for the inactivation of microorganisms, along with the monitoring of bacteria evolution using the plate counting method (Martínez-Pachón et al. 2021). Indeed, electrochemical technologies combined with UV radiation have been utilized to inactivate microorganisms such as fungal spores like *Penicillium polonicum* and *Trichoderma harzianum*, viruses like *Bacteriophage MS2*, as well as bacteria (e.g., *Enterococcus faecalis*, *Vibrio alginolyticus*, or even *E. coli*) (Herraiz-Carboné et al. 2022; Wan et al. 2022; Yoon et al. 2023).

The effectiveness of the coupling of the electrochemical process with UV, however, has been documented mainly in the binary UV/H_2_O_2_ combination (Herraiz-Carboné et al. 2022; Wan et al. 2022; Yoon et al. 2023). On the other hand, combination processes such as UV/ACS and UV/H_2_O_2_/ACS electrochemically assisted have been scarcely investigated so far, with no publications or patents having been found, according to a previous study (Espinosa-Barrera et al. 2024). This situation evidences a notable gap in the scientific-technological production for the study and application of these combinations (Espinosa-Barrera et al. 2024). Furthermore, the use of electrochemical processes with UVA-LEDs (at 365 nm) has only been assessed for bacteria inactivation of one microorganism (*E. coli*) (Chen et al. 2022; Jin et al. 2022).

Despite extensive research on the use of UV irradiation and electrochemical processes, either individually or in binary combinations, as disinfection methods, further research is needed to address the following aspects: i) The use of systems for disinfection combining long-wavelength UVA (λ > 390 nm, from low-cost LEDs) with electrogenerated H_2_O_2_ and ACS, using an undivided electrochemical flow cell with GDE cathodes and DSA anodes (UV-LEDs/GDE/DSA); ii) The implementation of a system with good disinfecting performance being operated at mild conditions of current density and supporting electrolyte concentration; iii) The evaluation of multiple bacteria in the same study; and iv) The application of flow cytometry techniques by photoelectrochemical treatment as an indicator of bacterial unviability.

Therefore, the main objective of this study was to assess the elimination of *E. coli* and *P. aeruginosa* (two relevant gram-negative bacteria) using a sequential recirculation electrochemical system, assisted with low-cost near-UV LEDs (UV-LEDs/GDE/DSA) at mild operational conditions. Three key aspects were considered herein: the inactivation efficiency, energy consumption, and the synergy of the photoelectrochemical combination. Furthermore, the viability of the bacteria was evaluated through flow cytometry as complementary analyses to the cultivability tests. Finally, a test was performed on a real irrigation water sample, a complex matrix that is continuously in contact with crops and can increase the propagation points of WDBs, which allowed to approach the evaluation of the efficiency of the UV-LEDs/GDE/DSA system in the disinfection of real waters under mild operating conditions.

## Materials and methods

### Microorganisms, culture media, and chemicals

Strains of *Escherichia coli* (ATCC 25922, Thermo Scientific™ Quanti-Cult™) and *Pseudomonas aeruginosa* (ATCC 10145, Thermo Scientific™ Microbiologics™) obtained from the basic sciences laboratory of the Universidad Antonio Nariño were implemented as model microorganisms for the disinfection processes. The reagents used were sodium chloride (NaCl ≥ 99.5%, Merck), potassium iodide (KI ≥ 99.5%, Merck), and ammonium heptamolybdate tetrahydrate ((NH_4_)_6_Mo_7_O_24_ ≥ 99.0%, Merck) analytical grade. Propidium iodide (PI, Invitrogen, ex/em 490/635 nm) molecular biology grade, and nutrient agar (ISO 6579, ISO 10273, ISO 19250, Panreac) microbiology quality.

### Reaction system

**Fig. 1:**
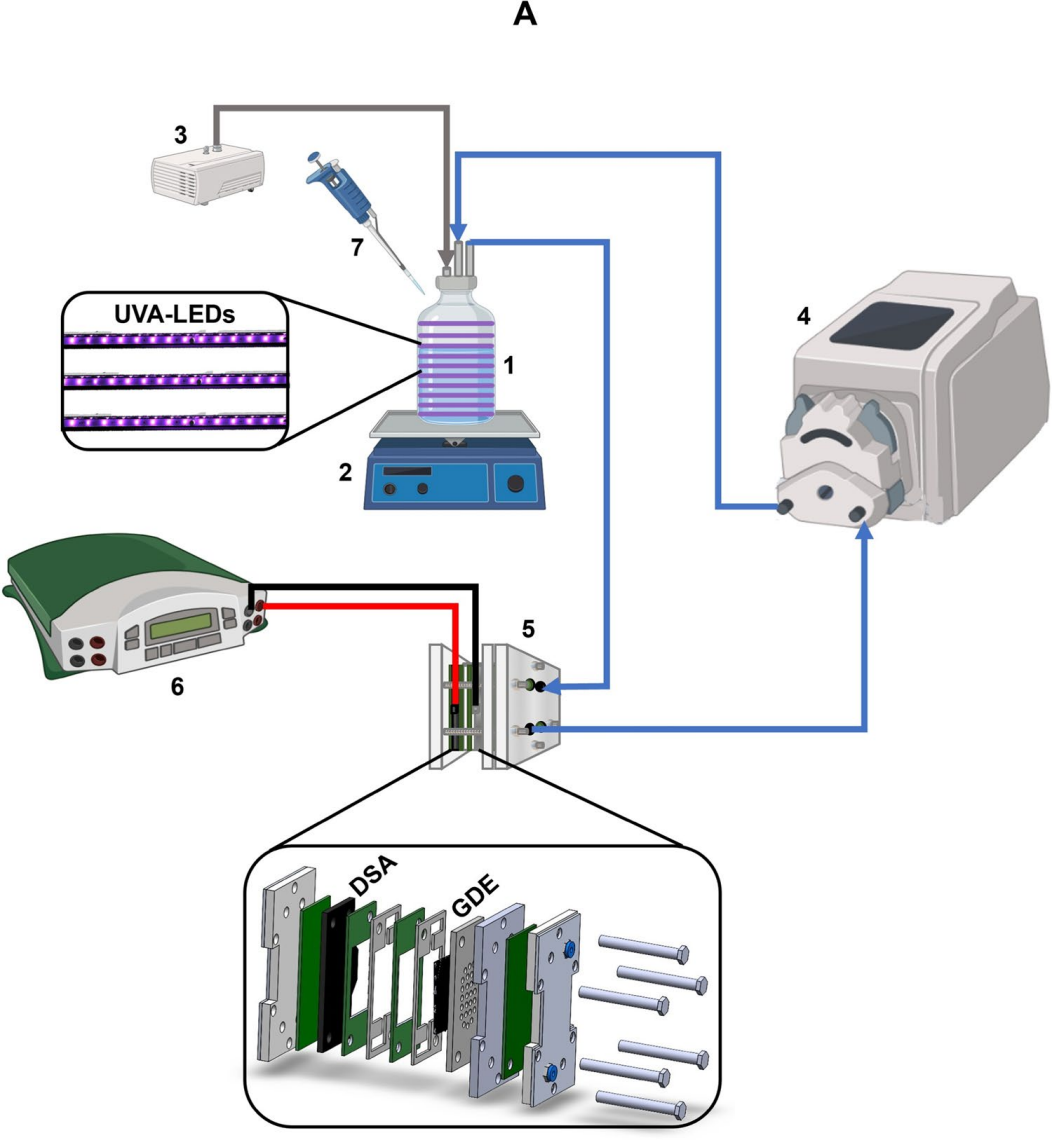

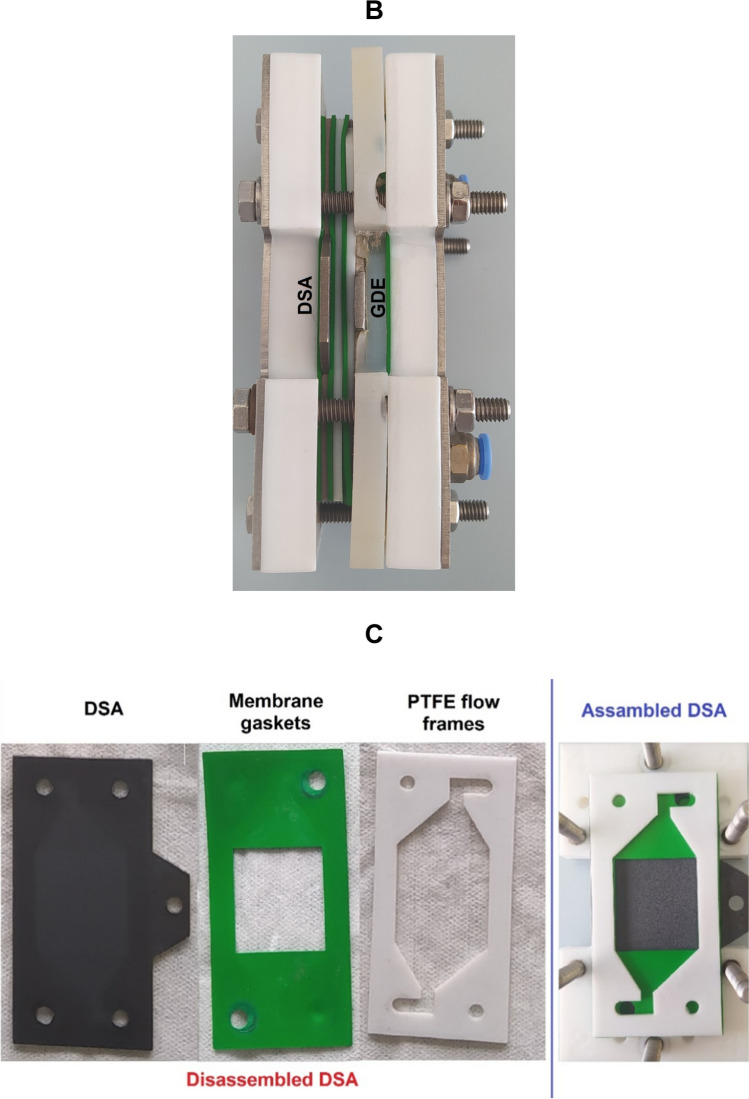

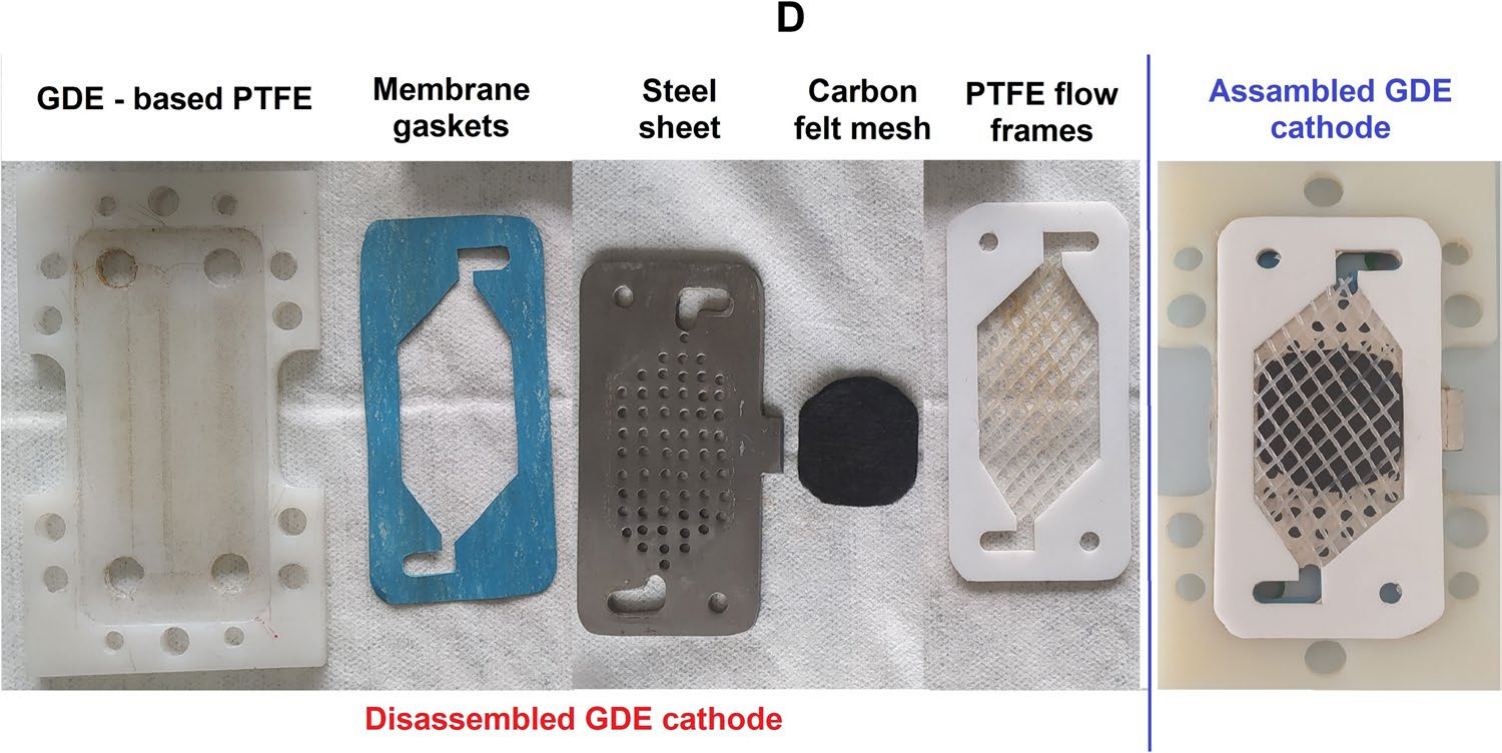
Experimental setup of the sequential recirculation electrochemical system assisted by UVA-LEDs (UV-LEDs/GDE/DSA). **A.** Schematic diagram showing: ***(1)*** Radiation tank with UVA-LED bands around it. ***(2)*** Stir plate. ***(3)*** Air compressor. ***(4)*** Peristaltic pump. ***(5)*** Flow electrochemical cell with GDE cathode and DSA anode. ***(6)*** Power supply. ***(7)*** Liquid samples. Blue line: water flow, gray line: airflow. **B*.*** Flow electrochemical cell assembled with: **C.** DSA (Left: Components for assembly, right: Assembled), and **D.** GDE cathode (Left: Components for assembly, right: Assembled)

The experiments were carried out in a recirculating electrochemical system assisted by UVA-LEDs at room temperature (Fig. 1). The reaction system was divided into two main components. The first main part is formed by 500 mL of the solution to be treated, which was irradiated with low-cost UVA-LED with emission between 395 and 400 nm (Model No: GL-UV-02, 2 W, 120 LEDs per meter), which were arranged around an irradiation tank, made of borosilicate 3.3 (number 1 in Fig. 1A). The target solution was divided into two matrices: i) A sterile NaCl solution containing an initial inoculum of 10^8^ CFU/mL of the model microorganisms (*E. coli* or *P. aeruginosa*), used in Sects. "Capability of the electrochemical system to generate disinfecting agents and inactivate bacteria", "Electrochemical system assisted with the UV-LEDs for inactivation of microorganisms and role of bacteria structure", and "Flow cytometry as a complementary technique to the monitoring of Gram-negative bacteria disinfection" of this study. ii) Real irrigation water obtained from a farm in the municipality of Mosquera, Colombia (4° 39′ 30.798" N 74° 13′ 48.559" W), where untreated stagnant wastewater is used for irrigation of crops of commercial interest, an important situation in the transmission of WDB (Delgado-Vargas et al. 2023). This was selected as a complex matrix model to evaluate the disinfection efficiency with the UV-LEDs/GDE/DSA system in a real matrix (Sect. "Potential of UV LED-assisted electrochemical system for disinfection of real irrigation water"). The physicochemical characterization of the water is described in a previous study (Delgado-Vargas et al. 2023).

The target solution was continuously stirred magnetically at a speed of 400 rpm on a VELP Scientifica ARECT magnetic stirrer to ensure proper mixing and transport of the reagents (number 2 in Fig. 1A). Before starting, the solution was aerated with an air compressor for 15 min to saturate the system with oxygen (number 3 in Fig. 1A). Table S1 (in the Supplementary material) presents the dissolved oxygen during the experiments. It should be mentioned that this did not affect the concentration of the two target microorganisms due to their facultative anaerobic nature, enabling them to proliferate oxidatively, using oxygen as a terminal electron acceptor, and being already adapted to high oxygen concentrations (as used herein), which allows them to regulate their metabolism in the presence of oxygen (Fu et al. 2015). Besides, no adjustments to the pH of the solution were made. Nevertheless, the pH slightly increased during the process, and it ranged between 6.41 and 7.17. The average initial and final pH of the treatments carried out by the system and subsystem are tabulated in Table S1. To avoid cross-contamination between experiments, pre- and post-treatment washings were performed (see Text S1). All experiments were conducted in triplicate.

The second main part of the reaction system is composed of the electrochemical microflow cell (number 5 in Fig. 1A), where the oxidizing species are generated. This electrochemical cell (ElectroCell Multi-Purpose Serial No.1670), is a press-type cell, i.e., its components (membrane gaskets, PTFE flow frames, GDE cathode, and DSA) are installed one after the other, and after the cell is closed and tightened with the screws on the periphery, the components are pressed together, leaving them fixed inside the cell (Fig. 1B) (Rodríguez et al. 2013). The cell components are 10 cm in high × 5 cm in length × 0.15 cm in width. A RuO_2_-doped TiO_2_-based DSA is installed in the electrochemical cell with a membrane gasket (to prevent water leakage) and a PTFE flow frame (which allows for a reaction booth on the electrode surface) (Fig. 1C). Similarly, a GDE cathode (carbon felt mesh-based) is installed with a PTFE base to fit the membrane gasket and a steel sheet (which will facilitate electron transfer by being directly connected to the power source), the carbon felt mesh is placed over the steel sheet (to have the electrodes on the mesh) and finally, a PTFE flow frame is placed (Fig. 1D). The electrodes have a reaction area of 10 cm^2^ each and have a distance of 0.5 cm between them. The system was run on direct current using a Cleaver Scientific—PowerPRO-300 power supply (number 6 in Fig. 1A).

The solution circulation between the two reaction system components is done by employing a Masterflex™ EW-77916–10 peristaltic pump (number 4 in Fig. 1A), recirculating solution between the two parts of the reactor at a flow rate of 241 mL/min. This configuration and flow rate allowed the microorganism to have a high dose of light exposure, which is relevant for disinfection in LED-based systems (Chen et al. 2017; Li et al. 2019). The connection between the two stages was made using silicone hoses (medical grade 1/6" × 1/4").

The sampling method was similar for oxidant measurements (Sect. "Measurement of oxidants"), plate count assays (Sect. "Plate count assays"), and bacterial non-viability measurements by flow cytometry (Sect. "Flow cytometry conditions"). A 100–1000 μL SelectPette micropipette was used to take aliquots from the radiation tank (number 7 in Fig. 1A) while the system was running. Aliquots were taken every minute until the end of treatment for all three cases, and the amount of sample varied depending on the measure to be performed (60 μL for oxidants analyses and 1 mL for plate counting and non-viability measurements).

In the aliquots, no quenching agent was added to neutralize the oxidizing species and terminate the reaction. This is because previous work has shown that the use of such agents (e.g., sodium bisulfite) can modify disinfection, which could lead to errors in the evaluations. Therefore, to avoid possible interferences, oxidant measurements, seeding of the samples, and measurement of non-viability were carried out immediately after taking the aliquot (Martínez-Pachón et al. 2021).

### Instrumentation and analytical measurements

#### Measurement of oxidants

The production of total oxidant species in the system was determined by the iodine metric method (detailed diagram in Fig. S1). Aliquots of 60 μL containing the oxidant were taken and placed in a spectrophotometer cell. Then 1350 μL of potassium iodide solution (0.1 mol/L) and 50 μL of ammonium heptamolybdate tetrahydrate solution (0.01 mol/L) were added. The sample was homogenized, and the absorbance of the solution was measured at a wavelength of 350 nm after 15 min in the dark. It should be noted that each experiment was repeated at least three times for sampling.

#### Plate count assays

The elimination of *E. coli* and *P. aeruginosa* was monitored by plate count (detailed diagram in Fig. S2). A 1 mL aliquot was taken from the irradiation tank and used to make serial decimal dilutions. 0.1 mL of these dilutions were seeded onto sterile nutrient agar Petri dishes, and the plates were incubated overnight at 37 °C. Subsequently, the number of culturable colony-forming units per milliliter present (CFU/mL) was counted.

To assess the regrowth of *E. coli* and *P. aeruginosa* after treatment, treated samples were stored in the dark for 48 h at room temperature. Then, 0.1 mL was reseeded on sterile nutrient agar plates for counting. The CFUs counted during regrowth experiments were considered damaged/inactivated bacteria that had previously failed to grow on the medium. Therefore, the amounts of bacteria during the regeneration test that exceeded the amounts of bacteria found during treatment were considered as repaired/reactivated bacteria.

It should be noted that all samples were cultured in triplicate and each experiment was repeated at least three times for sampling and culturing. Bacterial concentration was established in CFU/mL for each treatment time, and inactivation kinetics were determined by calculating log (N_t_/N_0_) vs. time, where N_0_ and N_t_ are the concentrations before and during the treatment, respectively (Martínez-Pachón et al. 2021).

#### Flow cytometry conditions

Cell viability was assessed using propidium iodide (PI), because when the bacterial cytoplasmic membrane is damaged, it allows PI to enter, which facilitates its binding to bacterial DNA and generates a fluorescence signal that is detected by flow cytometry. For this evaluation, 1 mL aliquots were taken from the irradiation tank (Fig. 1A). Then, 100 μL of the samples containing treated, untreated, and regenerated bacteria were taken and stained with PI (1 μg/mL). These samples were incubated for 10 min at room temperature in the dark and read on the flow cytometer (detailed diagram in Fig. S3). It should be noted that the collected aliquots were divided into three 250 μL Eppendorf tubes (each containing 100 μL of sample) and readings were performed in triplicate. In addition, each experiment was repeated at least three times for sampling. Untreated cells were used as the negative control, while 90% methanol-treated cells were used as the positive control.

Flow cytometry was performed with the BD Accuri™ C6 Plus instrument (BD Biosciences) equipped with a blue laser (488 nm, air-cooled, 20mW solid-state) and a 585/40, 670 LP filter specific for PI fluorescence. Diffracted light (related to cell surface: forward scatter FSC) and reflected light (related to granularity: side scatter SSC) from the blue laser, as well as PI, were collected. Data from 30,000 cells were collected using BD Accuri™ C6 software (version 1.0.264.21, BD Biosciences).

#### Identification of chlorinated disinfection by-products

To evaluate the potential formation of chlorinated disinfection by-products (DBPs) during treatment with a UV-LEDs/GDE/DSA system, an untreated water sample (T0) and two treated water samples with short (approximately 10 min, T10) and long (approximately 45 min, T45) exposure times were analyzed using a non-target approach based on gas chromatography coupled to high-resolution mass spectrometry. Water samples were extracted by solid-phase extraction (SPE) (adapted from Pitarch et al. 2007). Analysis was made with a Thermo Scientific™ Q Exactive™ GC hybrid quadrupole-Orbitrap mass spectrometer, controlled by Xcalibur 4.0 software (Thermo Scientific, Waltham, MA, USA). Compound Discoverer (Thermo Scientific, v3.3) was employed for data processing and untargeted analysis. More information can be found in Supplementary Materials (Text S3).

### Calculations

#### Electric energy consumption per order (E_EO_)

The electric energy required to achieve the elimination of the microorganism population (electric energy consumption per order of magnitude eliminated in Log_10_ in 1 m^3^, E_EO_) was obtained at one minute of treatment regardless of the system. The total E_EO_ (E_EO__/Total_) in terms of kWh/m^3^ is composed of the electric energy from the use of UV-LEDs (E_EO__/UV-LEDs_) and the electric energy from the use of the electrochemical cell (E_EO__/Cell_), which values were calculated using Eqs. 5 - 7 (Rehman et al. 2018; Tian et al. 2020).

5 $${E}_{EO/Total} = {E}_{EO/Cell} + {E}_{EO/UV-LEDs}$$

6 $${E}_{EO/Cell}=\frac{I * {E}_{cell}}{Q*Log(\frac{{C}_{f}}{{C}_{i}})}$$

7 $${E}_{EO/UV-LEDs}=\frac{P * t}{V*Log(\frac{{C}_{f}}{{C}_{i}})}$$

where, in terms of the flow electrochemical cell, I represent the applied current (kA), E_cell_ is the average cell voltage (in kV), and Q is the implemented flow rate (in m^3^/h). In terms of the radiation, P represents the power of the electronic energy input of the UV-LEDs used (in kW), V is the volume of the solution irradiated in the tank (in m^3^), and t is the radiation time (in h). For both cases, C_i_ and C_f_ are the initial and final concentrations of contaminants (e.i., CFU/mL), respectively.

#### Synergic index (φ)

The synergistic effect in the photo-electrochemical combination (UV-LEDs/GDE/DSA) and its corresponding binary subsystems (UV-LEDs/GDE, UV-LEDs/DSA, and GDE/DSA systems) was evaluated by comparing the synergy indexes (φ). These were calculated as the ratio of the inactivation rate (*k*) in a given combined system to the algebraic sum of the inactivation rates (*k*) in the subsystems involved (UV-LEDs, GDE, or DSA, Eq. 8) (Popova et al. 2021).

8 $$\varphi =\frac{ksubsystem\left(1\right) + ksubsystem\left(2\right)\dots + ksubsystem(n)}{kcombined\; system (1+2\dots +n)}$$

## Results and discussions

### Capability of the electrochemical system to generate disinfecting agents and inactivate *bacteria*

**Fig. 2:**
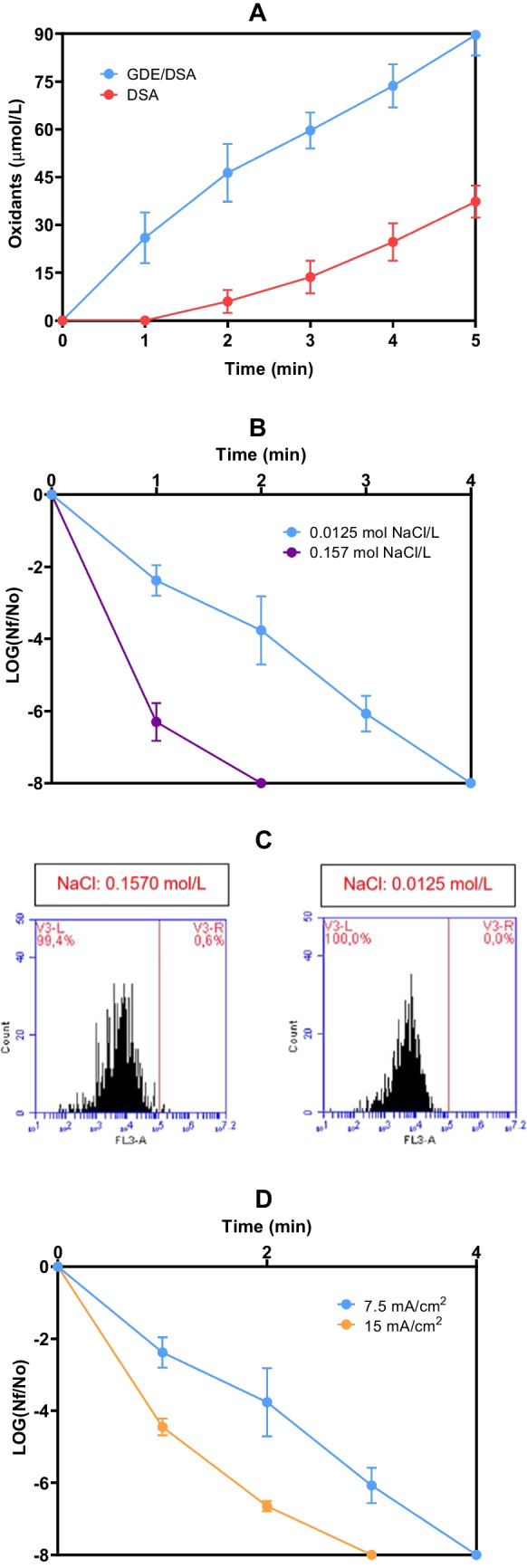
Controls on the generation of oxidants, inactivation, and unviability of *E. coli* under different conditions. **A.** Production of oxidants by the GDE/DSA electrochemical system without bacteria. System conditions: pH_initial_: 6.45, current density: 7.5 mA/cm^2^, flow: 241 mL/min, [NaCl]: 0.0125 mol/L, radiation source: N/A. **B.** Effect of the supporting electrolyte concentration on the inactivation of *E. coli* by the electrochemical system. System conditions: pH_initial_: 6.50, current density: 7.5 mA/cm^2^, flow: 241 mL/min, radiation source: N/A, *E. coli* concentration_initial_: 3 × 10^8^ CFU/mL. **C.** Salt stress controls of two NaCl concentrations on *E. coli* determined by flow cytometry (V3-L: histograms of alive bacteria). Conditions for evaluation: Volume of solution: 100 µL, *E. coli* concentration_initial_: 3 × 10^6^ CFU/mL, Temperature: 25.3 °C. **D.** Effect of the current density on the inactivation of *E. coli* by the GDE/DSA system. System conditions: pH_initial_: 6.49, flow: 241 mL/min, [NaCl]: 0.0125 mol/L, radiation source: N/A, *E. coli* concentration_initial_: 3 × 10^8^ CFU/mL. The bars represent the average of oxidant generation **(A)** and *E. coli* removal (**B** and **D**) in all treatments of the GDE/DSA system with a p ≤ 0.05 (n = 9, with three experiment replicates and three replicates measurement)

Initially, the electrochemical system's capability (without light) to produce disinfecting agents was tested without microorganisms (Figs. 2A and S4A). To evaluate the generation of ACS by the DSA anode, a stainless-steel electrode was used as the cathode, and the ultraviolet light remained turned off. To determine the combined ability of both DSA and GDE (i.e., GDE/DSA system) to produce oxidizing species, the experiments were also carried out without UV light (system setups for each control can be seen in detail in Table S1).

Figures 2A and S4A shows that the DSA alone produced ~ 37 µmol/L of oxidants after 5 min of electrolysis. Among these oxidants, the majority would be ACS, but a low concentration of H_2_O_2_ could also be generated on the surface of the cathode (stainless steel sheet, Table S1) following the same mechanism established for the GDE cathode, but with lower activity for the electrochemical production of H_2_O_2_ (Lim and Hoffmann 2019). Specifically, the generation of ACS is due to two situations. Firstly, it is attributed to the high efficiency of the DSA toward the oxidation of Cl^−^ to Cl_2_ (Eq. 2), followed by its subsequent dissolution in the aqueous medium (Cl_2(aq)_), which reduces losses in the form of gas (Ferreira de Melo et al. 2020; Martínez-Huitle et al. 2015). Secondly, the presence and predominance of the oxidizing species (i.e., HClO and ClO^−^) are regulated by the pH of the aqueous medium. At pH values greater than 3.0, Cl_2(aq)_ hydrolyzes to HClO (Eq. 3) with a rate constant (*k*_*1*_) of 22.3 s^−1^, while the reverse reaction involves a rate constant (*k*_*-1*_) of 4.3 × 10^4^ M^−2^ s^−1^, making favorable the formation of HClO (Eq. 5) (Deborde and von Gunten 2008). Moreover, at pH between 6.5 and 8.5, the HClO/ClO^−^ dissociation is incomplete (pKa for HOCl is 7.5), which causes the presence of both HClO and ClO^−^ in a chemical equilibrium (Antonelli et al. 2022; Ferreira de Melo et al. 2020; Martínez-Huitle et al. 2015).

In the DSA control system, the pH ranged from 6.69 to 7.17, and an average temperature of 25 °C, (Table S1). Then, these pH values close to neutral in the experiments allow HClO to prevail over ClO^−^. According to some authors and based on the ionization curve of HClO as a function of pH, it is estimated that HClO can be present at 88% at pH 6.69 (initial pH, Table S1) and 67% at pH 7.17 (final pH, Table S1) (Deborde and von Gunten 2008; Seymour et al. 2020). This can be beneficial for bacteria inactivation, as HClO is the ACS with the highest redox potential (E° = 1.49 V vs SHE), being recognized as one of the most powerful and fast-acting disinfectants (Antonelli et al. 2022; Wang et al. 2022).

This benefit for disinfection was observed by a reduction in the accumulation of ACS in the presence of 3 × 10^8^ CFU/mL *E. coli* (as a model microorganism), with a measured consumption of ~ 13 µmol/L ACS at 5 min after the start of disinfection (Fig. S4A). This suggests a direct interaction between bacteria and ACS during the disinfection process (Martínez-Pachón et al. 2019, 2021; Wang et al. 2022).

Meanwhile, the combined action of both DSA and GDE (without UV light) resulted in the production of ~ 90 µmol/L of oxidizing species in 5 min (Figs. 2A and S4A). These species consisted of ACS generated through the anodic action, as well as H_2_O_2_ produced via the cathodic reduction of oxygen (Eq. 3) (Delgado-Vargas et al. 2022; Martínez-Pachón et al. 2019), and there is even the possibility of the formation of singlet oxygen (^1^O_2_) through the interaction of HClO/OCl^−^ with H_2_O_2_ (Eqs. 9–10) (Barazesh et al. 2015).

9 $${\text{HClO}+\text{H}}_2{\text{O}}_2\rightarrow\text{HCl}+{\text{H}}_2\text{O}+{{}^1\mathrm O}_2$$

10 $$\text{OCl}^-+{\text{H}}_2{\text{O}}_2\rightarrow\text{Cl}^-+{\text{H}}_2\text{O}+{{}^1\mathrm O}_2$$

On the other hand, a similar effect on disinfection of both cathode GDE (H_2_O_2_) and anode DSA (ACS) generated oxidants was observed. During *E. coli* removal, comparable consumption was recorded in the GDE/DSA system (20 µmol/L total oxidants at 5 min of disinfection, Fig. S4A) concerning the DSA system alone (~ 13 µmol/L ACS less than that observed without bacteria). These results highlight the high capacity of the electrochemical system to generate several species such as ACS, H_2_O_2_, and ^1^O_2_, in addition to demonstrating their action by attacking bacteria and thus facilitating the water disinfection process (Ersoy et al. 2019; Spuhler et al. 2010).

In the combined DSA and GDE system, the pH oscillated between 6.52 (initial pH) and 6.61 (final pH), with an average temperature of 25 °C (Table S1). These slight changes in pH values, in this GDE/DSA system or the DSA control, can be attributed mainly to a buffering effect caused by the dynamic equilibrium known as the chlorine-water equilibrium, based on the formation and dissociation of HClO. These equilibria have been studied in NaCl solutions extensively, especially in the context of ACS electrogeneration in aqueous media (Deng et al. 2023). In the specific systems addressed in this work, the buffer keeps pH close to 7, favoring their applicability in disinfection. As mentioned above, at our experimental pH ranges, the presence of HClO prevails, which is the most efficient disinfectant of the three ACS due to its highest redox potential (E° = 1.49 V vs SHE). Besides, the buffer effects ensure the presence of this species throughout the disinfection process (Antonelli et al. 2022; Wang et al. 2022).

On the other hand, the inactivation of *E. coli* (as the model microorganism), by the electrochemical component alone (i.e., GDE/DSA), was tested. The operation of the electrochemical system is determined by two relevant parameters, the current density and the concentration of the supporting electrolyte (Martínez-Sánchez et al. 2022). Therefore, the effect of these two parameters on the efficiency of the GDE/DSA system was evaluated. Trials were performed to assess *E. coli* inactivation at two different concentrations of NaCl (0.1570 and 0.0125 mol/L) and two different current densities (7.5 and 150 mA/cm^2^).

The concentrations of the supporting electrolyte were selected considering several factors. A minimum concentration of 0.0125 mol/L of NaCl was chosen because this amount of supporting electrolyte provides the minimum conductivity of ~ 1440 μS/cm required for the operation of the electrochemical cell (data obtained using the conductivity model, Text S2) (Tangphant et al. 2014) according to the distance between the two electrodes (0.5 cm). Moreover, it must be considered that the obtained conductivity value (1440 µS/cm) by using 0.0125 mol/L of NaCl belongs to the typical range of conductivity reported for some real wastewater (500–1500 µS/cm) (Ma et al. 2018). Although the conductivity of real wastewater depends on other ions such as Ca^2+^, Mg^2+^, CO_3_^2−^, HCO_3_^−^, or SO_4_^2−^; Cl^−^ ions are usually present at considerable concentrations in liquid effluents and natural water. Hence, the use of NaCl allows us to simulate the role of Cl^−^ in real wastewater (de Moura et al. 2014; Martínez-Huitle et al. 2015).

The NaCl concentration of 0.1570 mol/L (which is the concentration of the saline solution widely used as a diluent to adjust the turbidity of bacterial cell suspensions) (Eaton 1852; Ohtomo and Saito 2001), was chosen for the following reasons: i) to reduce the potential hyperpermeability of the cell membrane (saline stress in bacteria), preventing the entry of oxidizing species into the bacteria and avoiding false positives in cell inactivation or viability. Also, this concentration serves as a negative control for saline stress or damage to microorganisms by disinfecting agents (Arense et al. 2010; Ohtomo and Saito 2001); ii) under constant conditions of current density and electrode distance in the system, this NaCl concentration allows us to get an efficient electron transfer between the electrodes, since it is the concentration that provides a conductivity close to the highest limit value allowed in this system (~ 18,000 µS/cm) according to the data obtained from the conductivity model (Text S2) (Ma et al. 2018; Tangphant et al. 2014); iii) this NaCl amount allows obtaining a wide range of difference (~ 13-folds) compared to the low concentration (0.0125 mol/L); and iv) the NaCl concentration of 0.1570 mol/L provide us 18,000 µS/cm, which is close to the conductivity of water matrices such as some brine wastewater, seawater, or contaminated estuaries that can contain pathogenic bacteria (Ma et al. 2018; Nguyen-Sy et al. 2021). Although the concentration of 0.0125 mol NaCl/L may induce salt stress on the model bacteria, no false positives were observed in either the plate count or flow cytometry technique (Figs. 2B and C, respectively). On the contrary, the recovery and increase in proliferation were observed after the adaptation period at this NaCl concentration (Ohtomo and Saito 2001). At this point, the permeability phenomenon is reduced, as evidenced by minimal PI reagent mobility to enter the bacteria in the flow cytometry tests (Fig. 2C) (Arense et al. 2010; Ohtomo and Saito 2001).

At the two NaCl concentrations, the bacteria inactivation (Fig. 2B) and energy consumption (E_EO/Cell_) were determined (Table 1). The rate constant of *E. coli* inactivation decreased from 6.705 to 2.186 Log_10_/min when 0.1570 and 0.0125 mol/L of supporting electrolyte concentration were used, respectively. Increasing the concentration of NaCl can lead to a higher concentration of ACS (Eqs. 2–4) (Flores-Terreros et al. 2022). When the sodium chloride concentration was increased from 0.0125 mol/L to 0.1570 mol/L, the total oxidant concentration rose from approximately 90 µmol/L to 165 µmol/L (Fig. S4B)., resulting in faster inactivation of *E. coli*. Specifically, complete inactivation of the bacteria was achieved in 2 min with 0.1570 mol/L NaCl, compared to 4 min with 0.0125 mol/L NaCl (Fig. 2B).

**Table 1:** Inactivation rate constants (*k*) and electric energy consumption (E_EO/Cell_) for the inactivation of *E. coli* by the DSA/GDE system at different supporting electrolyte concentrations and current densities

Current density (mA/cm^2^)	NaCl (mol/L)	*k* (Log_10_/min)	Kinetic formula	R^2^	E_EO/Cell_ (kWh/m^3^ order)
7.5	0.0125	2.186	Log = -2.186*t + 8.067	0.9876	5.43 × 10^–6^
7.5	0.1570	6.705	Log = -6.705*t + 8.374	0.9999	2.32 × 10^–6^
15.0	0.0125	3.328	Log = -3.328*t + 7.857	0.9560	1.36 × 10^–5^

*All the data in the table represent the average of three replicated experiments and three replicated measurements, *p* ≤ 0.05 (*n* = 9)

Moreover, the E_EO/Cell_ only changed from 2.32 × 10^–6^ kWh/m^3^ order in Log_10_ for 0.157 mol/L to 5.43 × 10^–6^ kWh/m^3^ order in Log_10_ for 0.0125 mol/L. This indicates that, from a practical standpoint, opting for the lowest addition of NaCl is advisable, as the system operates efficiently at this concentration (which represents the minimum required for viable system operation). Furthermore, the system already demonstrates sufficient efficiency at this concentration, with fewer reagent consumption and significantly close E_EO/Cell_ values, resulting in no significant difference in electrical energy consumption.

The effect of NaCl concentration on *E. coli* inactivation was also examined at two current densities (7.5 and 15.0 mA/cm^2^) (Table 1 and Fig. 2D). It was observed that the *E. coli* inactivation rate and E_EO/Cell_ were higher when 15 mA/cm^2^ was applied compared to the results obtained at 7.5 mA/cm^2^. The current density is a key parameter in the electrochemical processes because it controls the generation of oxidizing species and the rate of oxidation reactions (Martínez-Sánchez et al. 2022). A higher value of *k* (3.328 Log_10_/min) was obtained at 15 mA/cm^2^, in contrast to the value obtained with 7.5 mA/cm^2^ (2.186 Log_10_/min), due to the high electron quantity available for the production of disinfecting species (e.g., H_2_O_2_, ACS, and ^1^O_2_) able to inactivate *E. coli* (Çelebi et al. 2015). This was evidenced in Fig. S4C, where the concentration of total oxidants at 5 min of treatment was 1.8 times higher when using 15 mA/cm^2^ (~ 162 µmol/L of total oxidants), compared to the concentration obtained with 7.5 mA/cm^2^ (~ 90 µmol/L).

A comparison of the *E. coli* evolution under the two current densities shows that 7.5 mA/cm^2^ requires a slightly longer inactivation time (4 min) (Fig. 2D). Despite the extended inactivation time at 7.5 mA/cm^2^, its E_EO/Cell_ value (5.43 × 10^–6^ kWh/m^3^ order in Log_10_) was significantly lower than the value calculated for 15 mA/cm^2^ (1.36 × 10^–5^ kWh/m^3^ order in Log_10_), indicating a higher efficiency inactivation using 7.5 mA/cm^2^. The decrease in efficiency at the higher current density can be associated with parasite reactions, such as the O_2_ evolution at the anode (Ferreira de Melo et al. 2020; Hakizimana et al. 2022) which consume some electrons, resulting in energy wastage and circuit heating, increasing energy consumption and operating costs (Şahinkaya 2013).

On the contrary, with a lower current density, such as 7.5 mA/cm^2^, a suitable production of oxidizing species is obtained efficiently using the electroactive sites on the electrodes, thus leading to a lower E_EO/Cell_ for the complete bacteria inactivation (Hakizimana et al. 2022). Therefore, based on the above results on the inactivation of *E. coli*, the conditions 7.5 mA/cm^2^ and 0.0125 mol NaCl/L were used in the subsequent experiments, as they were determined to be the minimum viable conditions for the efficient operation of the system.

### Electrochemical system assisted with the UV-LEDs for inactivation of microorganisms and role of *bacteria* structure

**Fig. 3:**
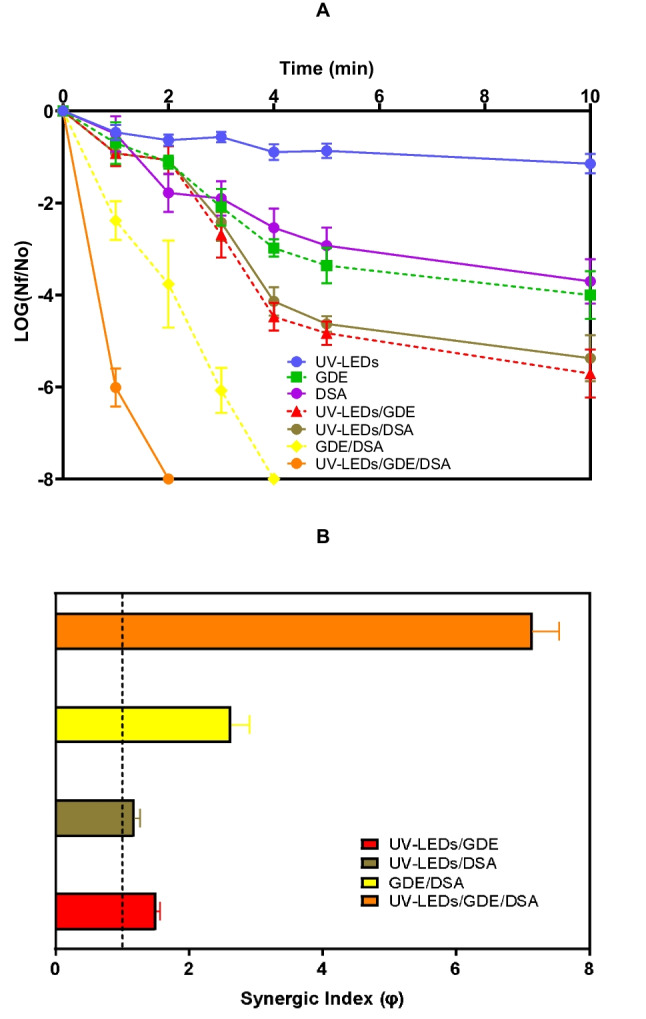
Inactivation of *E. coli* **A.** Comparison of bacteria inactivation by individual subsystems (UV-LEDs, GDE, and DSA), binary subsystems (UV-LEDs/GDE, UV-LEDs/DSA, and GDE/DSA), and the coupled system (UV-LEDs/GDE/DSA), followed by plate counting. **B.** Synergistic Indexes (φ) for the inactivation of *E. coli*. System conditions: Average pH_initial_: 6.50, Average pH_final_: 6.79, current density: 7.5 mA/cm^2^, flow: 241 mL/min, [NaCl]: 0.0125 mol/L, radiation source: UV-LEDs, *E. coli* concentration_initial_: 3 × 10^8^ CFU/mL. The bars represent the average in *E. coli* removal **(A)** and the calculation of synergy during these removals **(B)** in all treatments with different systems at *p* ≤ 0.05 (*n* = 9, with three experiment replicates and three replicates measurement)

After establishing the suitable operational conditions for *E. coli* inactivation, the effects of the electrochemical system (GDE/DSA) assisted with UV-LEDs were assessed. Figure 3A presents the *E. coli* evolution in the UV-LEDs/GDE/DSA system, and the comparison of this process with its corresponding subsystems (i.e., GDE/DSA, UV-LEDs/DSA, UV-LEDs/GDE, DSA, GDE, and UV-LEDs). Remarkably, complete inactivation of *E. coli* was observed after only 2 min of treatment by implementing the combined UV-LEDs/GDE/DSA system, which is faster than that obtained by the electrochemical system alone (GDE/DSA).

The dual subsystems (UV-LEDs/DSA, UV-LEDs/GDE, and GDE/DSA) and the single subsystems (DSA, GDE, and UV-LEDs) were less efficient than the UV-LEDs/GDE/DSA process for the bacterial inactivation (Fig. 3A). Indeed, after 10 min of treatment, UV-LEDs, DSA, and GDE achieved ~ 1.0, 3.1 and 3.2 Log-units reduction, respectively. In these last three single subsystems, the disinfecting action can be associated with the photo-inactivating action of UV light (395–400 nm) (Yin et al. 2013), the attacks of electrogenerated ACS (Ersoy et al. 2019; Wang et al. 2022), and H_2_O_2_ (Spuhler et al. 2010) to *E. coli*, correspondingly.

The efficiency of the subsystems (GDE/DSA, UV-LEDs/DSA, and DSA) and the complete system (UV-LEDs/GDE/DSA) in bacterial inactivation was confirmed to be due to the oxidants generated, and the incidence of UV-LEDs radiation (Fig. S4D). It was observed that the systems with the highest total oxidant production at 10 min were the GDE/DSA subsystem and the complete UV-LEDs/GDE/DSA system, being 2.29 times higher than the single subsystems DSA and the dual subsystem UV-LEDs/DSA. This difference is attributed to the contribution of H₂O₂ generated by the GDE cathode (Eq. 1, Fig. S4D).

Furthermore, it was confirmed that UV-LEDs have mainly a direct photoinactivating effect on bacteria since irradiation between 395 and 400 nm does not induce photolysis of the generated oxidizing species that, under other conditions, could originate additional radicals such as hydroxyl (OH·) and chlorine radical (Cl·) (Moreno-Andrés et al. 2023; Pai and Wang 2022; Espinosa-Barrera et al. 2024). In this study, this does not occur because the main active chlorine species (HClO/ClO^−^) absorb UV light in the range of 200 to 375 nm, with maximum absorption centered at 236 nm for HOCl (ε = 101 ± 2 M^−1^ cm^−1^) and 292 nm for ClO^−^ (ε = 365 ± 8 M^−1^ cm^−1^), while H₂O₂ has its maximum absorption at 254 nm (ε = 18.6 ± 2 M^−1^ cm^−1^) (Stefan 2017: Luo et al. 2015). This results in similar concentrations of oxidants generated in the GDE/DSA subsystem when compared to the full UV-LEDs/GDE/DSA system, and between the DSA subsystem and the UV-LEDs/DSA (Fig. S4D). These results indicate a consistent contribution of each subsystem (GDE, DSA, UV-LEDs) to the complete UV-LEDs/GDE/DSA system in bacterial inactivation.

The evolution of oxidant production and bacterial inactivation is not sufficient to justify the combinations. To better support this, the synergy (φ) was determined for the binary subsystems (i.e., UV-LEDs/DSA or UV-LEDs/GDE) and the complete system (i.e., UV-LEDs/GDE/DSA) (Fig. 3B). A φ value greater than 1 indicates synergistic system, φ equal to 1 represents an additive process, whereas a φ value lower than 1 means that we have an antagonistic system (Popova et al. 2021).

In the *E. coli* inactivation (Fig. 3B), the UV-LEDs/GDE (red bar), UV-LEDs/DSA (brown bar), and GDE/DSA (yellow bar) systems presented φ values of 1.51, 1.18, and 2.63, respectively. These synergies are due to the simultaneous generation and action of different disinfecting agents (Lineback et al. 2018; Zeng et al. 2020). For instance, in the UV-LEDs/GDE system, it is possible the photo-inactivation (Yin et al. 2013) and the disinfecting action of electrogenerated H_2_O_2_, thus leading to synergistic effects compared to GDE or UV-LEDs acting solely. Likewise, in the case of the UV-LEDs/DSA, in addition to the photo-inactivation of bacteria, we can have the direct effects of electrogenerated ACS on the microorganism (Fig. S4A and S4D), thus forming a synergistic system. In turn, the GDE/DSA system, which promotes the generation and action of ACS and H_2_O_2_ (Delgado-Vargas et al. 2022; Giannakis et al. 2016; Martínez-Pachón et al. 2021), can also induce the formation in situ of ^1^O_2_ (Eqs. 9–10), which is a strong disinfecting agent (Lu et al. 2022).

The complete system (UV-LEDs/GDE/DSA) has a synergistic index of 7.15 for the inactivation of *E. coli* (Fig. 3B, orange bar). This φ value is 2 to 6 times higher than that presented by the binary combined systems (UV-LEDs/GDE, UV-LEDs/DSA, and GDE/DSA). The high synergy in the complete process is attributed to the simultaneous effects provided by all its composing subsystems (i.e., the action of the UVA irradiation, and the attacks of ACS, H_2_O_2_, or ^1^O_2_ formed in situ from the reaction of the electrogenerated ACS and H_2_O_2_) (Martínez-Pachón et al. 2021; Tian et al. 2020; Wang et al. 2020). Since the UV-LEDs/GDE/DSA system had the greatest potential to obtain complete inactivation of *E. coli* (Fig. 3A), this treatment was applied to deal with another gram-negative bacterium (*P. aeruginosa*) but with different morphological/structural characteristics.

*P. aeruginosa* has a single polar flagellum, while *E. coli* has a peritrichous arrangement of flagella. In addition, the peptidoglycan layer in *P. aeruginosa* is not as strongly bonded to the outer membrane as it is in *E. coli* (i.e., the peptidoglycan layer in *P. aeruginosa* is more separated from the outer membrane) (Yao et al. 1999). Moreover, the outer membrane of *E. coli* has higher permeability than that of *P. aeruginosa* (Nikaido et al. 1991).

**Fig. 4:**
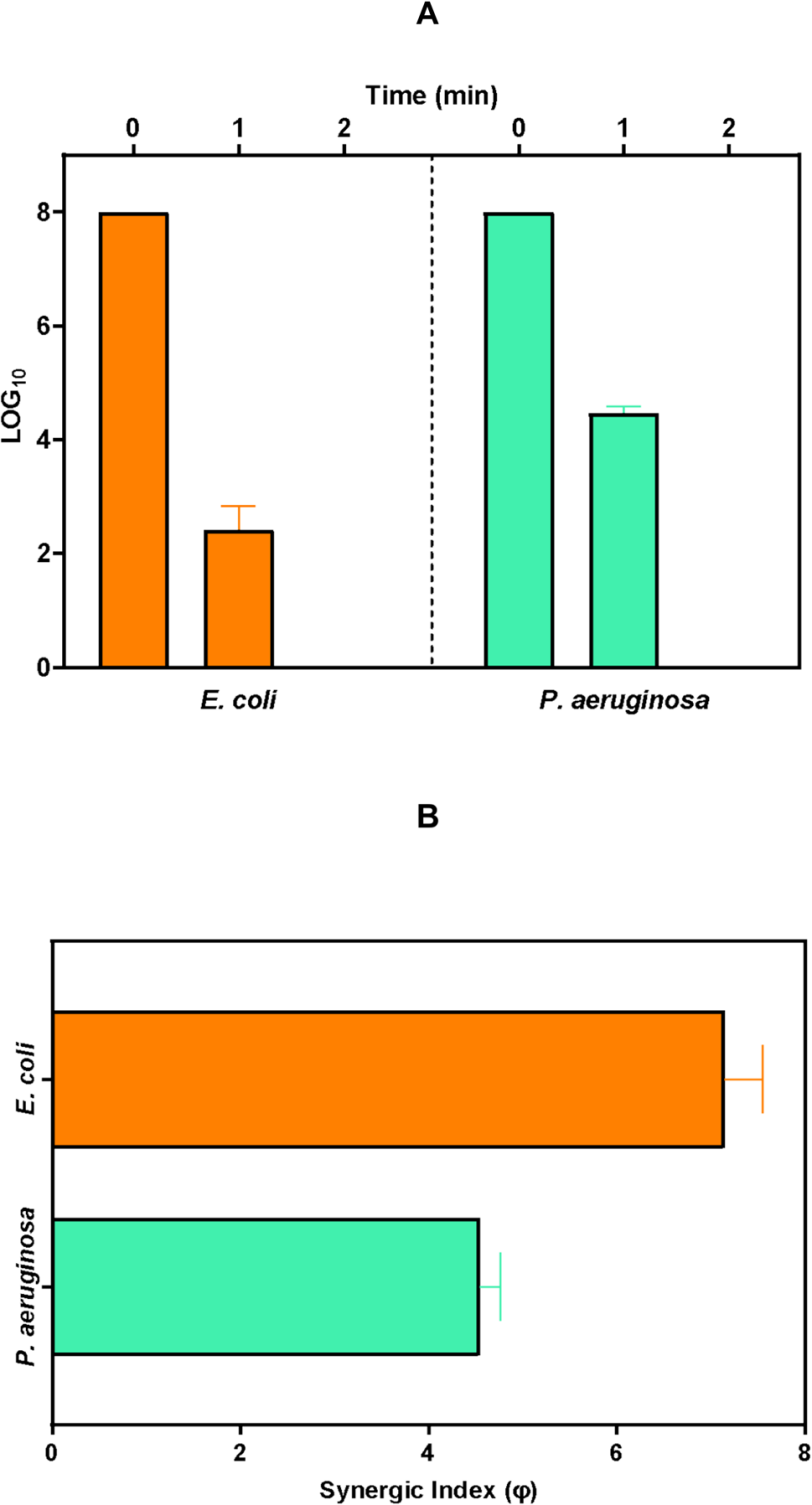
Comparison of inactivation of the two gram-negative bacteria by the photo-electrochemical treatment (UV-LEDs/GDE/DSA). **A.** inactivation of *E. coli* and *P. aeruginosa* after 1 min of treatment. **B.** Synergistic Index (φ) for the inactivation of both gram-negative bacteria by the UV-LEDs/GDE/DSA system. System conditions: Average pH_initial_: 6.59, average pH_final_: 6.74, current density: 7.5 mA/cm^2^, flow: 241 mL/min, [NaCl]: 0.0125 mol/L, radiation source: UV-LEDs, bacteria concentration_initial_: 3 × 10^8^ CFU/mL. The bars represent the average bacteria removal **(A)** and the calculation of synergy during these removals **(B)** in all treatments with the UV-LEDs/GDE/DSA system at *p* ≤ 0.05 (*n* = 9, with three experiment replicates and three replicates measurement)

Figure 4A compares the removal of both gram-negative bacteria by the UV-LEDs/GDE/DSA system. It can be noted that after 1 min of treatment, the system achieved a higher inactivation of *E. coli* (6 Log units) compared to *P. aeruginosa* (~ 4.7 Log units. Additionally, the UV-LEDs/GDE/DSA system has a synergistic index of 4.54 for the elimination of *P. aeruginosa,* while the φ value for *E. coli* was 7.15 (Fig. 4B). These results were attributed to the structural/morphological differences between the two microorganisms.

It is hypothesized that *P. aeruginosa* exhibits greater resistance due to the absence of a peritrichous arrangement of flagella, and its outer membrane is more separated from the peptidoglycan layer compared to *E. coli.* This suggests that the external attacks of electrogenerated radicals have a higher probability of affecting more structural components of the latter microorganism. Moreover, due to *E. coli* having higher permeability than *P. aeruginosa* (Nikaido et al. 1991), the former microorganism could be penetrated more easily by electrogenerated H_2_O_2_ or HClO, thus making it more susceptible to faster and more synergistic disinfection.

The hypothesis was supported by the identification of membrane damage and changes in the permeability of both microorganisms using flow cytometry. The propidium iodide (PI) can diffuse inside the cell and bind to nucleic acids only when the membrane is damaged, resulting in increased membrane permeability and higher PI intensity (Ohtomo and Saito 2001; Wang et al. 2022). Damaging the membrane is an important mechanism for bacterial inactivation. Before starting the treatment for both microorganisms, the average percentage of viable cells was 98.4% for *E. coli* and 97.8% for *P. aeruginosa* (Fig. S5). After 1 min of treatment, a higher proportion of *E. coli* cells shifted to an intermediate state compared to *P. aeruginosa*, ~ 69.5% and ~ 54.1%, respectively (Fig. S5). This indicates that a larger number of *E. coli* cells experienced significant membrane damage, leading to increased permeability and greater penetration of oxidizing species (Nikaido et al. 1991). This finding is consistent with the previously mentioned inactivation results (Fig. 4A), where *P. aeruginosa* showed greater resistance to the UV-LEDs/GDE/DSA system compared to *E. coli*. However, it is important to note that after 2 min, most of the population of both microorganisms became non-viable (dead cells, Fig. S5), representing 94.7% and 91.9% of dead cells for *E. coli* and *P. aeruginosa*, respectively, while the remaining percentage in both cases consisted in cells still in the intermediate state. Thus, the complete inactivation and non-viability of the microorganisms were observed after 2 min of treatment by the UV-LEDs/GDE/DSA system.

It is important to mention that the UV-LEDs/GDE/DSA system was also able to achieve the complete inactivation of *P. aeruginosa* after 2 min of treatment. In addition to evaluating disinfection efficiency and synergy in the elimination of the two bacteria, the E_EO/Total_ was calculated for the complete system (UV-LEDs/GDE/DSA) and its component subsystems (Table 2). The complete system was one of the systems with the lowest E_EO/Total_ (1.13 × 10^–2^ kWh/m^3^ order in Log_10_ for *E. coli* and 1.55 × 10^–2^ kWh/m^3^ order in Log_10_ for *P. aeruginosa*). These E_EO/Total_ values were 4 to 100 times lower than those obtained for the subsystems UV-LEDs or the combined systems (UV-LEDs/GDE and UV-LEDs/DSA). This is because the UV-LEDs subsystem and the combined systems with UV-LEDs exhibited low disinfection efficiencies, resulting in longer treatment times to achieve complete inactivation, as described in the literature (Serna-Galvis et al. 2020; Song et al. 2019).

**Table 2:** E_EO/Total_ for the inactivation of gram-negative bacteria by the subsystems and the combined system

Bacteria	E_EO/Total_ (kWh/m^3^ order)						
	UV-LEDs	GDE	DSA	UV-LEDs/GDE	UV-LEDs/DSA	DSA/GDE	UV-LEDs/GDE/DSA
*E. coli*	1.26 × 10^–1^	3.14 × 10^–5^	3.47 × 10^–5^	6.81 × 10^–2^	5.48 × 10^–2^	5.43 × 10^–6^	1.13 × 10^–2^
*P. aeruginosa*	1.03 × 10	7.35 × 10^–5^	6.05 × 10^–5^	1.29 × 10^–1^	1.39 × 10^–1^	5.96 × 10^–6^	1.55 × 10^–2^

*All the data in the table represent the average of three replicated experiments and three replicated measurements, *p* ≤ 0.05 (*n* = 9)

On the contrary, the lower E_EO/Total_ values of the combined GDE/DSA system (5.43 × 10^–6^ kWh/m^3^ order in Log_10_ for *E. coli* and 5.96 × 10^–6^ kWh/m^3^ order in Log_10_ for *P. aeruginosa*) compared to those obtained with UV-LEDs/GDE/DSA is explained considering the first one does not have a radiation source (Popova et al. 2021). However, the addition of the light source accelerated the bacteria inactivation (Fig. 3A).

### Flow cytometry as a complementary technique to the monitoring of Gram-negative *bacteria* disinfection

**Fig. 5:**
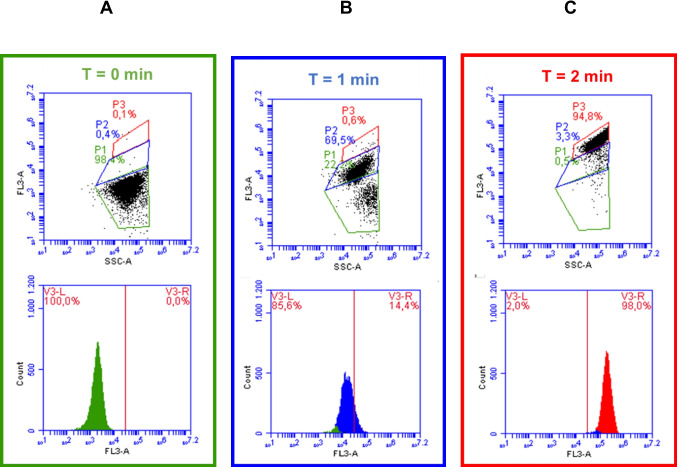
Evolution of the results of flow cytometry and PI staining during the treatment of *E. coli* using the UV-LEDs/GDE/DSA system (*upper: dot plots and bottom: histograms*). **A.** Non-treated bacteria. **B.** *E. coli* treated for 1 min. **C.** *E. coli* treated for 2 min. System conditions: Average pH_initial_: 6.50, average pH_final_: 6.56, current density: 7.5 mA/cm^2^, flow: 241 mL/min, [NaCl]: 0.0125 mol/L, radiation source: UV-LEDs, *E. coli* concentration_initial_: 3 × 10^6^ CFU/mL. Conditions for evaluation: Volume of solution: 100 µL, Temperature: 25.3 °C

The cultivability assay through plate counting assumes that only the bacteria capable of reproducing themselves are viable cells (Ohtomo and Saito 2001; Wang et al. 2022). However, there is a problem with these measurements because microbial cells are not classified as "live/dead". The suggested states of cryptobiotic, dying, and dormant have been proposed over the years (Martínez-Sánchez et al. 2022), and complementary techniques are required to overcome this concern. Therefore, in this study, flow cytometry combined with PI staining was used to assess the type and extent of damage caused by the photo-electrochemical process to *E. coli* as the target bacterium (Fig. 5).

Flow cytometry allowed us to identify live cells (appearing in the green quadrant, P1), dead cells (red quadrant, P3), as well as intermediate and unlabeled states in P2 in the blue quadrant (Fig. 5). It must be mentioned that the PI staining test indicates non-viable cells. The PI cannot cross the membrane of alive cells, and PI staining denotes a loss of viability (Giannakis et al. 2016). Firstly, the viability of the initial *E. coli* was confirmed by flow cytometry.

Figure 5A shows that at 0 min of treatment, most of the bacterial population (P1: 98.4%) is viable, indicating that the microorganisms before treatment have their cell membrane intact (as also displayed at the left side of the histogram (V3-L) in Fig. 5A). After 1 min of *E. coli* treatment by the UV-LEDs/GDE/DSA system, the most population of microorganisms changed to an intermediate state (Fig. 5B, see quadrant P2 and its corresponding histogram), which is associated with too strongly injured but not dead bacteria. Meanwhile, after 2 min of treatment, a significant decrease in the percentage of microorganisms in the intermediate state, and a high increase of dead bacteria were observed in quadrant P3 (Fig. 5C). This indicates that more than 94% of *E. coli* suffered membrane damage, resulting in bacterial unviability (Rehman et al. 2018; de Moura et al. 2014).

The membrane damage and subsequent bacteria unviability promoted by the complete system (UV-LEDs/GDE/DSA) can be rationalized by considering the simultaneous presence of various disinfecting factors within the system. A wide variety of species are all acting in the system, i.e., ACS from the Cl^−^ oxidation on the DSA (Eqs. 2–4), the H_2_O_2_ from the O_2_ reduction on GDE (Eq. 1), ^1^O_2_ from ACS and H_2_O_2_ reaction (Eqs. 9–10), and reactive oxygen species formed by the interaction of bacteria with the UV-LEDs. Importantly, the pre-treatment dissolved oxygen concentrations (between 6.45 and 6.69 mg O_2_/L, Table S1) did not influence the initial concentration of the microorganisms, attributing the membrane damage and the complete inactivation of the gram-negative bacteria to the presence of these oxidizing species.

In the case of the ACS, the disinfection mechanism is mainly based on attacking the external membrane of the bacteria, oxidizing cellular components, increasing permeabilization of the cytoplasmic membrane, but also internal actions such as uncoupling of electron chains, and deactivation of enzymes (Martínez-Pachón et al. 2021; Scialdone et al. 2021). Then, the action of ACS also explains the inactivation and unviability shown in Fig. 3A and S6 (see purple line). On the other hand, the H_2_O_2_, which is electrogenerated on the surface of the GDE, can directly oxidize the components of the bacterial outer membrane (Lineback et al. 2018; Zeng et al. 2020). Moreover, O_2_ and H_2_O_2_ in the system facilitate the internal acceptance of electrons, increasing intracellular disinfecting ROS within the bacteria (Giannakis et al. 2016; Sun et al. 2019). These aspects support the removal of up to 3.8-Log of *E. coli* and an unviability of 7% after 10 min of treatment by the GDE subsystem (green line in Fig. 3A and S6). Meanwhile, the combined action of electrogenerated ACS and H_2_O_2_ produces ^1^O_2_, which may induce further oxidative damage outside the bacteria through lipid peroxidation of the membrane (Prasad et al. 2019; Yin et al. 2013), adding to the bacteria inactivation resulting from the attacks of the electrogenerated ACS and H_2_O_2_ (yellow line in Fig. 3A and S6).

The UV-LEDs (UVA having wavelengths of 395—400 nm) used in the system can inactivate bacteria by a photosensitized process. During this process, naturally occurring photosensitizers (PS) such as porphyrins and flavins in the microbial cells are excited by the UVA light, producing an excited triplet state of the PS. The interaction of the long-lived excited triplet state of the PS with the ground state of molecular oxygen (also in a triplet state) results in the formation of reactive oxygen species (ROS) such as ^1^O_2_ through energy transfer, and superoxide radical anion and hydroxyl radicals through electron transfers. These ROS can chemically attack a very wide range of biomolecules, thus affecting the viability of the bacteria (Prasad et al. 2019; Yin et al. 2013). These effects of the UVA light were associated with inactivation of up to 1.1-Log in *E. coli* and 1% unviability at 10 min of treatment (blue line in Fig. 3A and S6).

On the other hand, the ROS generated by the action of UV-LEDs on the bacterial PS can also attack membrane proteins, allowing other oxidant species (e.g., H_2_O_2_ and ACS) to penetrate the bacteria (Pai a Wang 2022; Wallace et al. 2019; Wang et al. 2019), and enhancing the intracellular damages, thus achieving 5.5-Log and 5.3-Log removal of *E.coli* at 10 min of treatment, with a unviability of 60% and 56%, respectively (as shown by the red and brown lines in Fig. 3A and S6).

Finally, in the UV-LEDs/GDE/DSA system is integrated the action of H_2_O_2_, ACS, ^1^O_2_, and photo-generated ROS (Delgado-Vargas et al. 2022; Giannakis et al. 2016; Martínez-Pachón et al. 2021, 2019). Hence, this led to the bacteria suffering irreparable damage to the cell membrane allowing the PI penetration and facilitating its binding to bacterial DNA (Comninellis and Chen 2010) (Fig. 5C), which is consistent with a complete (8-Log), reduced viability (> 94% dead cells), and fast (only 2 min of treatment) inactivation of the target bacteria (orange line in Fig. 3A and S6). These results denote that the UV-LEDs/GDE/DSA system is very efficient for the elimination of bacteria.

### Potential of UV LED-assisted electrochemical system for disinfection of real irrigation water

Considering the high efficiency of the UV-LEDs/GDE/DSA sequential recirculation electrochemical system in a controlled environment (Sects. "Capability of the electrochemical system to generate disinfecting agents and inactivate bacteria" to "Flow cytometry as a complementary technique to the monitoring of Gram-negative bacteria disinfection"), its performance was evaluated under relevant conditions using a real irrigation water sample as a first approach to its application in the disinfection of real matrices. An irrigation water sample was selected from the municipality of Mosquera-Colombia since this water comes from stagnant wastewater in artificial canals of the irrigation district 'La Ramada', which receives water from the Bogotá River, untreated wastewater from Bogotá, and nearby municipalities. This water is used without prior treatment for commercial crops, such as lettuce, which poses a risk of crop contamination and spread of WBDs (Chen et al. 2021; Delgado-Vargas et al. 2023).

The treatment of the irrigation water sample with the UV-LEDs/GDE/DSA system was performed under the minimum viable conditions previously established for optimum efficiency (7.5 mA/cm^2^ and 0.0125 mol NaCl/L). During the treatment (60 min), the elimination of culturable bacteria in CFU/mL, oxidant generation in µmol/L, and total organic carbon (TOC) removal in percentage terms were monitored.

**Fig. 6:**
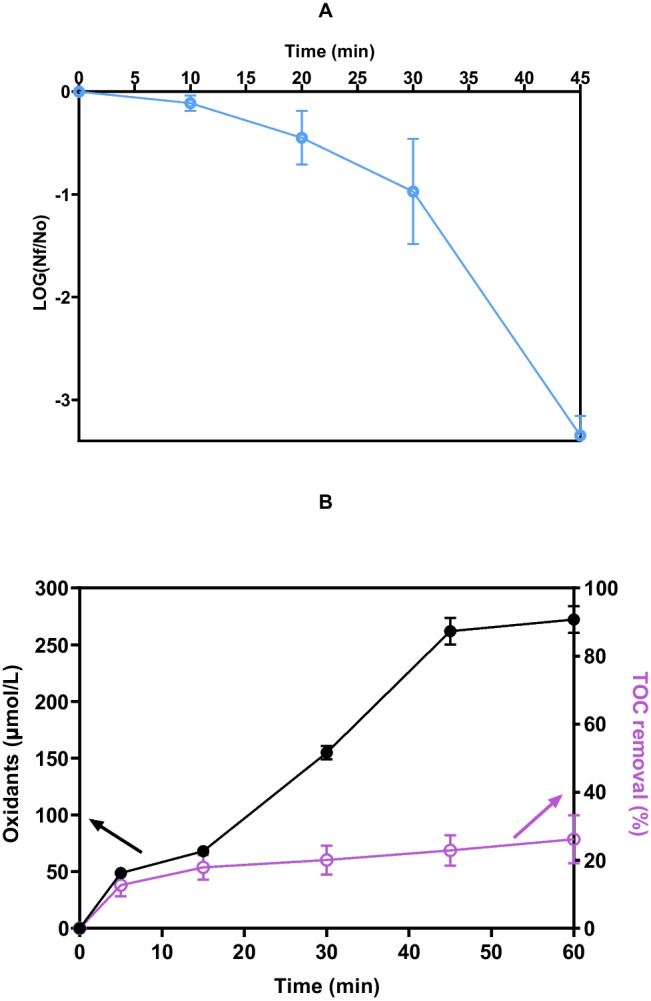
Evolution in the disinfection of irrigation water samples using the UV-LEDs/GDE/DSA system. **A.** Elimination of culturable bacteria. **B.** Production of total oxidants and TOC removal. System conditions: Average pH_initial_: 6.80, average pH_final_: 7.05 current density: 7.5 mA/cm^2^, flow rate: 241 mL/min, [NaCl]: 0.0125 mol/L, radiation source: UV LED, bacteria concentration_initial_: 2.3 × 10^3^ CFU/mL. Bars represent average culturable bacteria removal **(A)**, total oxidant production, and percent TOC removal **(B)** across treatments (*p* ≤ 0.05, *n* = 6 with two experiment replicates and three measurement replicates)

Figure 6A shows that complete inactivation of the initial culturable bacteria (~ 10^3^ CFU/mL) with the UV-LEDs/GDE/DSA system required 45 min of treatment. A significant increase in the time required to achieve the same disinfection efficiency observed under controlled conditions (complete inactivation of 8-Log in 2 min of treatment, orange line Fig. 3A) was observed. Considering the initial conditions of the irrigation water, which showed a TOC of 70.63 mg/L, turbidity of 69 UNT, and concentrations of chemical micropollutants, specifically pharmaceutically active compounds detected between 10 and 1000 ng/L in our previous work (Delgado-Vargas et al. 2023), the interaction of oxidants (H_2_O_2_, ACS, and ^1^O_2_) and UVA radiation with the target bacteria was hindered due to competition with the other non-target contaminants in the disinfection (Martínez-Pachón et al. 2022; Espinosa-Barrera et al. 2024). This situation affected the efficiency of the treatment, despite having achieved complete elimination of the bacteria.

This increased competition for the oxidizing species generated is evidenced by the higher consumption of these species during irrigation water treatment (Fig. 6B). At 5 min, the production was ~ 49 µmol/L, being 1.42 times lower than that observed when removing 8-Log *E. coli* under controlled conditions (Fig. S4A). Despite the additional consumption of oxidants, a progressive increase in the accumulation of total oxidants (H_2_O_2_, ACS, and ^1^O_2_) was observed after 15 min of treatment, reaching ~ 272 µmol/L at 60 min. The increased accumulation of oxidants together with UVA radiation allowed a ~ 26% removal of the initial TOC at 60 min of treatment, resulting in a residual TOC of 51.9 mg/L. The residual organic matter after treatment can be attributed to compounds such as humic and fulvic acids, along with short-chain organic acids, as well as micropollutants (e.g., hygiene products, pesticides, pharmaceuticals, etc.).These compounds are resistant to treatment and are challenging to achieve complete mineralization in these systems (Martínez-Pachón et al. 2022; Shi et al. 2023).

One of the most relevant problems when disinfecting waters with high organic matter content is the formation of DBP, mainly due to the reaction of chlorinated disinfectants with organic matter of natural and anthropogenic origin. These by-products can be cytotoxic, teratogenic, mutagenic, and genotoxic, associated with human health issues (Li et al. 2022). Although it was not the primary objective of this work, as it was focused on bacteria inactivation, we performed a tentative identification of potential chlorinated DBPs formed along the system UV-LEDs/GDE/DSA treatment to have a more comprehensive picture of the process applied to real water samples. Analysis was made by GC-high-resolution mass spectrometry following a non-target approach. In the real irrigation waters subjected to this treatment, up to eight chlorinated DBPs were identified, as shown in Table 3. The identification process of these species is explained in detail in Supplementary material (Text S3 and Fig. S7). Only those chlorinated compounds that presented higher abundances in T10 and T45 to T0 were considered in the list.

**Table 3:** Identified DBPs by GC-QOrbitrap MS in wastewater samples treated with the UV-LEDs/GDE/DSA at different exposure times

NIST Lib Hit Name	Formula	Id.*Level	T0	T10	T45	Hazard
1,2,3,4-tetrachlorocyclopenta-1,3-diene	C_5_H_2_Cl_4_	2		✓	✓✓	*NA*
Trichlorophenol	C_6_H_3_Cl_3_O	3		✓	✓✓	*Irritant, Toxic to aquatic life with long-lasting effects*
2-(4-chlorophenoxy) ethan-1-ol	C_8_H_9_ClO_2_	2		✓	✓✓	*Corrosive, Irritant*
Trichloroaniline	C_6_H_4_Cl_3_N	3		✓	✓✓	*Acute toxic, Irritant, Suggestive evidence of carcinogenic potential, Toxic to aquatic life with long-lasting effects*
Tri(2-chloroethyl) phosphate	C_6_H_12_Cl_3_O_4_P	2			✓	*Irritant, Known human carcinogen, Toxic to aquatic life with long-lasting effects*
Bis(1-chloro-2-propyl)(3-chloro-1-propyl)phosphate	C_9_H_18_Cl_3_O_4_P	3	✓	✓✓	✓✓✓	*NA*
Bis(3-chloro-1-propyl)(1-chloro-2-propyl)phosphate	C_9_H_18_Cl_3_O_4_P	3	✓	✓✓	✓✓✓	*NA*
tris(2-chloro-1-methylethyl) phosphate (TCIPP)	C_9_H_18_Cl_3_O_4_P	3	✓	✓✓	✓✓✓	*Irritant, Harmful to aquatic life with long-lasting effects*

*Id. Level: Confidence in the identification based on the five-level classification proposed for HRMS-based identifications in environmental samples (Schymanski et al. 2014). T0 = non-treated wastewater; T10 and T45 = 10 min and 45 min of treatment. The tick symbol (✓) indicates the presence of the compound in the sample; ✓✓and ✓✓✓ represent a higher relative abundance of the compound in the sample. NA = not available; Hazard information was obtained from U.S. EPA’s CompTox Chemicals Dashboard (https://comptox.epa.gov/dashboard/)

The compound 1,2,3,4-tetrachlorocyclopenta-1,3-diene was tentatively identified in the treated samples. The mechanism of halocyclopentadienes formation in chlorinated waters and their toxicity have been reported in the scientific literature (Li, et al. 2022). Although other halocyclopentadienes are known to be extremely toxic for mammals and aquatic organisms, there is no information about toxicity for 1,2,3,4-tetrachlorocyclopenta-1,3-diene. Chlorophenols are another group of DBPs known to be formed during chlorination treatments (Önnby et al. 2018). In this work, an isomer, or a mixture of isomers, of trichlorophenol was tentatively identified in treated water. These species deserve special attention due to their potential environmental hazard and ability to transform into trihalomethanes or haloacetic acids (Önnby et al. 2018).

Other halobenzenes tentatively identified during the treatment included 2-(4-chlorophenoxy) ethan-1-ol and an isomer, or a mixture of isomers, of trichloroaniline. The latter compound has suggestive evidence of carcinogenic potential. Lastly, the formation of four chlorinated organophosphate flame retardants was observed. Tri(2-chloroethyl) phosphate was found at 45 min of treatment, and three isomers with the molecular formula C_9_H_18_Cl_3_O_4_P were present in all samples, with a significant increase as the treatment progressed.

Based on the results obtained in the first phase application of the sequential recirculating GDE/DSA electrochemical system combined with low-cost UV-LEDs for the disinfection of contaminated water, it is imperative to explore alternatives or modifications that maintain or improve disinfection efficiencies while mitigating post-treatment risks, such as DBPs formation.

Alternatives to consider include: i). Use of LEDs in the UVC and UVB ranges to generate other oxidizing species and facilitate photolysis of contaminants that contribute to the reduction of organic matter and pathogenic microorganisms. ii). Reduction of current densities to minimize the production of ACS and thus the possible generation of DBPs, without compromising the disinfection efficiency of the water. iii). Implementation of sequential phased recirculation, starting with electrochlorination using DSA followed by electroperoxidation using GDE, acting as a post-chlorination treatment.

Neither single subsystems (GDE, DSA, UV-LEDs) nor dual combinations (GDE/DSA, UV-LEDs/GDE, UV-LEDs/DSA) are proposed due to their lower disinfection efficiency, as observed in this study (see Fig. 3A and S6). Furthermore, these systems are similarly likely to generate DBPs compared to electrochlorination alone (DSA alone and UV-LEDs/DSA), due to the comparative ACS production of the subsystems with that observed in the full UV-LEDs/GDE/DSA system (Figure S4D).

Also, it is crucial to perform specific economic cost analyses and life cycle analysis for the UV-LEDs/GDE/DSA system to more accurately assess its applicability. These aspects will be addressed in future work, as they were not the focus of the present study.

## Conclusions

The sequential electrochemical system with low-cost UV-LEDs (UV-LEDs/GDE/DSA), a system not previously reported in the literature, has demonstrated outstanding efficacy in water disinfection. In 2 min of treatment, complete inactivation of model microorganisms was obtained, with low energy consumptions (1.13 × 10^–2^ kWh/m^3^ order for *E. coli* and 1.55 × 10^–2^ kWh/m^3^ order for *P. aeruginosa*) and high synergy (7.15 for *E. coli* and 4.54 for *P. aeruginosa*). These energy consumptions are lower, and the φ are higher compared to single subsystems (UV-LEDs, GDE, and DSA) and binary systems (UV-LEDs/GDE, UV-LEDs/DSA and GDE/DSA). The combination of various disinfectants (ACS, H_2_O_2_, ^1^O_2_, ROS, and UVA irradiation) in the UV-LEDs/GDE/DSA system enhances effective inactivation mechanisms, such as damage to bacterial membrane, proteins, and DNA. This allows obtaining a highly efficient system in the disinfection of the model microorganisms studied, despite implementing the system in the minimum conditions required for efficient operation (7.5 mA/cm^2^ and 0.0125 mol NaCl/L).

When evaluating the system in a relevant environment using irrigation water samples, results with potential for application of the system were observed, including inactivation of culturable bacteria by 3-log after 45 min of treatment and a reduction of initial TOC by approximately 26% in 60 min of treatment. However, the tentative formation of DBPs was detected by GC-HRMS, suggesting the need to address strategies to mitigate these post-treatment risks. Consequently, the study indicates the good potential of the UV-LEDs/GDE/DSA system for future applications in the disinfection of water contaminated with relevant microorganisms. However, several alternatives and modifications are identified that could be explored to minimize post-treatment adverse effects without compromising disinfection efficiencies, pointing out important areas for further and future research.

## Supplementary Information

Below is the link to the electronic supplementary material.

Supplementary file1 (DOCX 3156 KB)

## Data Availability

All data generated or analyzed during this study are included in this published article [and its supplementary information files].

## References

[CR1] Antonelli R, Malpass GRP, da Silva MGC, Vieira MGA (2022) Photo-assisted electrochemical degradation of ciprofloxacin using DSA® anode with NaCl electrolyte and simultaneous chlorine photolysis. J Water Process Eng 47:102698. 10.1016/j.jwpe.2022.102698

[CR2] Arense P, Bernal V, Iborra JL, Cánovas M (2010) Metabolic adaptation of Escherichia coli to long-term exposure to salt stress. Process Biochem 45(9):1459–1467. 10.1016/j.procbio.2010.05.022

[CR3] Ayadi A, Proietto F, Jaouadi M, Hamzaoui AH, Galia A, Scialdone O (2023) Treatment of aqueous solutions of oxytetracycline by different electrochemical approaches: anodic oxidation, pressurizedelectro-Fenton and oxidation by electrogenerated active chlorine. J Chem Technol Biotechnol 98(8):1955–1963. 10.1002/jctb.7413

[CR4] Barazesh JM, Hennebel T, Jasper JT, Sedlak DL (2015) Modular advanced oxidation process enabled by cathodic hydrogen peroxide production. Environ Sci Technol 49(12):7391–7399. 10.1021/acs.est.5b01254 26039560 10.1021/acs.est.5b01254 PMC4473729

[CR5] Bavasso I, Poggi C, Petrucci E (2020) Enhanced degradation of paracetamol by combining UV with electrogenerated hydrogen peroxide and ozone. J Water Process Eng 34:101102. 10.1016/j.jwpe.2019.101102

[CR6] Çelebi MS, Oturan N, Zazou H, Hamdani M, Oturan MA (2015) Electrochemical oxidation of carbaryl on platinum and boron-doped diamond anodes using electro-Fenton technology. Sep Purif Technol 156(3):996–1002. 10.1016/j.seppur.2015.07.025

[CR7] Chaplin BP (2018) Advantages, disadvantages, and future challenges of the use of electrochemical technologies for water and wastewater treatment. In: Martínez-Huitle CA, Rodrigo MA, Scialdone O (ed) *Electrochemical water and wastewater treatment*, 1st edn. Butterworth-Heinemann, pp 451–494. 10.1016/B978-0-12-813160-2.00017-1

[CR8] Chen J, Loeb S, Kim J-H (2017) LED revolution: fundamentals and prospects for UV disinfection applications. Environ Sci: Water Res Technol 3(2):188–202. 10.1039/C6EW00241B

[CR9] Chen Y, Duan X, Zhou X, Wang R, Wang S, Ren N, Ho S-H (2021) Advanced oxidation processes for water disinfection: Features, mechanisms and prospects. Chem Eng J 409:128207. 10.1016/j.cej.2020.128207

[CR10] Chen X, Chen Z, Lin C-Y, Chen R, Huang P, Jin Y (2022) Water disinfection by the UVA/electro-Fenton process under near neutral conditions: Performance and mechanisms. Chemosphere 308(Pt 3):136488. 10.1016/j.chemosphere.2022.136488 36152825 10.1016/j.chemosphere.2022.136488

[CR11] Coha M, Farinelli G, Tiraferri A, Minella M, Vione D (2021) Advanced oxidation processes in the removal of organic substances from produced water: Potential, configurations, and research needs. Chem Eng J 414:128668. 10.1016/j.cej.2021.128668

[CR12] Comninellis C, Chen G (2010) Electrochemistry for the Environment. In: Henry Bergmann ME (ed) Drinking Water Disinfection by In-line Electrolysis: Product and Inorganic By-Product Formation, 1st edn. Springer, New York, pp 163–204. 10.1007/978-0-387-68318-8

[CR13] Cordeiro-Junior PJM, Lobato Bajo J, de Lanza MRV, Rodrigo Rodrigo MA (2022) Highly efficient electrochemical production of hydrogen peroxide using the GDE technology. Ind Eng Chem Res 61(30):10660–10669. 10.1021/acs.iecr.2c01669 35941851 10.1021/acs.iecr.2c01669 PMC9354083

[CR14] de Moura DC, de Araújo CKC, Zanta CLPS, Salazar R, Martínez-Huitle CA (2014) Active chlorine species electrogenerated on Ti/Ru0.3Ti0.7O2 surface: Electrochemical behavior, concentration determination and their application. J Electroanal Chem 731:145–152. 10.1016/j.jelechem.2014.08.008

[CR15] Deborde M, von Gunten U (2008) Reactions of chlorine with inorganic and organic compounds during water treatment-Kinetics and mechanisms: a critical review. Water Res 42(1–2):13–51. 10.1016/j.watres.2007.07.025 17915284 10.1016/j.watres.2007.07.025

[CR16] Delgado-Vargas CA, Espinosa-Barrera PA, Villegas-Guzman P, Martínez-Pachón D, Moncayo-Lasso A (2022) An efficient simultaneous degradation of sulfamethoxazole and trimethoprim by photoelectro-Fenton process under non-modified pH using a natural citric acid source: study of biodegradability, ecotoxicity, and antibacterial activity. Environ Sci Pollut Res Int 29(28):42275–42289. 10.1007/s11356-021-17751-5 34993786 10.1007/s11356-021-17751-5

[CR17] Delgado-Vargas CA, Barreneche-Vasquez JS, Cógua NG, Botero-Coy AM, Hernández F, Martínez-Pachón D, Moncayo-Lasso A (2023) Optimization and application of a continuous flow photo-electro-Fenton system for the removal of pharmaceutical active compounds detected in irrigation water of Bogotá-Savanna (Colombia) Crops. J Environ Chem Eng 11(5):111030. 10.1016/j.jece.2023.111030

[CR18] Deng Z, Xu S, Liu C, Zhang X, Li M, Zhao Z (2023) Stability of dimensionally stable anode for chlorine evolution reaction. Nano Res. 10.1007/s12274-023-5965-7

[CR19] Eaton TD (1852) Saline solution. Notes Queries s1-VI(165):612–612. 10.1093/nq/s1-VI.165.612d

[CR20] Ersoy ZG, Dinc O, Cinar B, Gedik ST, Dimoglo A (2019) Comparative evaluation of disinfection mechanism of sodium hypochlorite, chlorine dioxide and electroactivated water on Enterococcus faecalis. LWT 102:205–213. 10.1016/j.lwt.2018.12.041

[CR21] Espinosa-Barrera PA, Gómez-Gómez M, Vanegas J, Machuca-Martinez F, Torres-Palma RA, Martínez-Pachón D, Moncayo-Lasso A (2024) Systematic analysis of the scientific-technological production on the use of the UV, H2O2, and/or Cl2 systems in the elimination of bacteria and associated antibiotic resistance genes. Environ Sci Pollut Res 31(5):6782–6814. 10.1007/s11356-023-31435-2 10.1007/s11356-023-31435-2 PMC10821820 38165540

[CR22] Fakhkhari P, Tajeddin E, Azimirad M, Salmanzadeh-Ahrabi S, Abdi-Ali A, Nikmanesh B, Alebouyeh M (2022) Involvement of Pseudomonas aeruginosa in the occurrence of community and hospital acquired diarrhea, and its virulence diversity among the stool and the environmental samples. Int J Environ Health Res 32(1), 61–71. 10.1080/09603123.2020.1726300 10.1080/09603123.2020.1726300 32073302

[CR23] Ferreira de Melo J, Medeiros de Araújo D, Ribeiro da Silva D, Martinez Huitle CA, Villegas-Guzman P (2020) Applicability of electrochemical technology for treating a real petrochemical effluent by electro-generated active chlorine species. Int J Electrochem Sci 15(10):10262–10275. 10.20964/2020.10.66

[CR24] Ferro G, Guarino F, Castiglione S, Rizzo L (2016) Antibiotic resistance spread potential in urban wastewater effluents disinfected by UV/H2O2 process. Sci Total Environ 560–561:29–35. 10.1016/j.scitotenv.2016.04.047 10.1016/j.scitotenv.2016.04.047 27093120

[CR25] Flores-Terreros RR, Serna-Galvis EA, Navarro-Laboulais J, Torres-Palma RA, Nieto-Juárez JI (2022) An alternative approach to the kinetic modeling of pharmaceuticals degradation in high saline water by electrogenerated active chlorine species. J Environ Manage 315:115119. 10.1016/j.jenvman.2022.115119 35500483 10.1016/j.jenvman.2022.115119

[CR26] Fu H, Yuan J, Gao H (2015) Microbial oxidative stress response: Novel insights from environmental facultative anaerobic bacteria. Arch Biochem Biophys 584:28–35. 10.1016/j.abb.2015.08.012 26319291 10.1016/j.abb.2015.08.012

[CR27] Giannakis S, Ruales-Lonfat C, Rtimi S, Thabet S, Cotton P, Pulgarin C (2016) Castles fall from inside: Evidence for dominant internal photo-catalytic mechanisms during treatment of Saccharomyces cerevisiae by photo-Fenton at near-neutral pH. Appl Catal B 185:150–162. 10.1016/j.apcatb.2015.12.016

[CR28] Hakizimana I, Zhao X, Wang C, Zhang C, Pan S, Li Y, Liu N (2022) Effective Degradation of Amoxicillin by Multi-Stage Flow-Through Electrochemical System Using Porous Electrodes. SSRN Electron J. 10.2139/ssrn.4083101

[CR29] Herraiz-Carboné M, Cotillas S, Lacasa E, Cañizares P, Rodrigo MA, Sáez C (2022) Depletion of ARGs in antibiotic-resistance Klebsiella, Pseudomonas and Staphylococcus in hospital urines by electro and photo-electro disinfection. J Water Process Eng 49:103035. 10.1016/j.jwpe.2022.103035

[CR30] Jin Y, Chen Z, Chen X, Huang P, Chen X, Ding R, Chen R (2022) The drinking water disinfection performances and mechanisms of UVA-LEDs promoted by electrolysis. Journal of Hazardous Materials, 435, 129099. 10.1016/j.jhazmat.2022.129099 10.1016/j.jhazmat.2022.129099 35650736

[CR31] Li X, Cai M, Wang L, Niu F, Yang D, Zhang G (2019) Evaluation survey of microbial disinfection methods in UV-LED water treatment systems. Sci Total Environ 659:1415–1427. 10.1016/j.scitotenv.2018.12.344 31096352 10.1016/j.scitotenv.2018.12.344

[CR32] Li J, Aziz MT, Granger CO, Richardson SD (2022) Halocyclopentadienes: An Emerging Class of Toxic DBPs in Chlor(am)inated Drinking Water. Environ Sci Technol 56(16):11387–11397. 10.1021/acs.est.2c02490 35938673 10.1021/acs.est.2c02490

[CR33] Lim J, Hoffmann MR (2019) Substrate oxidation enhances the electrochemical production of hydrogen peroxide. Chem Eng J Lausanne, Switzerland: 1996 374:958–964. 10.1016/j.cej.2019.05.165 10.1016/j.cej.2019.05.165 PMC6686209 31624468

[CR34] Lineback CB, Nkemngong CA, Wu ST, Li X, Teska PJ, Oliver HF (2018) Hydrogen peroxide and sodium hypochlorite disinfectants are more effective against Staphylococcus aureus and Pseudomonas aeruginosa biofilms than quaternary ammonium compounds. Antimicrob Resist Infect Control 7:154. 10.1186/s13756-018-0447-5 30568790 10.1186/s13756-018-0447-5 PMC6298007

[CR35] López AV, López KH, Giannakis S, Benítez N (2017) Effect of reactor material and its reuse on photo-Fenton process efficiency at near-neutral pH: Alterations in E. coli inactivation and resorcinol degradation kinetics in water. J Photochem Photobiol, A 344:228–237. 10.1016/j.jphotochem.2017.04.019

[CR36] Lu X, Zhou X, Qiu W, Wang Z, Cheng H, Zhang H, … Ma J (2022) Singlet oxygen involved electrochemical disinfection by anodic oxidation of H2O2 in the presence of Cl−. Chemical Engineering Journal, 446, 136871. 10.1016/j.cej.2022.136871

[CR37] Luo C, Ma J, Jiang J, Liu Y, Song Y, Yang Y, Wu D (2015) Simulation and comparative study on the oxidation kinetics of atrazine by UV/H2O2, UV/HSO5− and UV/S2O82−. Water Res 80:99–108. 10.1016/j.watres.2015.05.019 10.1016/j.watres.2015.05.019 25996757

[CR38] Ma P, Ma H, Sabatino S, Galia A, Scialdone O (2018) Electrochemical treatment of real wastewater. Part 1: Effluents with low conductivity. Chem Eng J 336:133–140. 10.1016/j.cej.2017.11.046

[CR39] Martínez-Huitle CA, Rodrigo MA, Sirés I, Scialdone O (2015) Single and coupled electrochemical processes and reactors for the abatement of organic water pollutants: A critical review. Chem Rev 115(24):13362–13407. 10.1021/acs.chemrev.5b00361 26654466 10.1021/acs.chemrev.5b00361

[CR40] Martínez-Pachón D, Espinosa-Barrera P, Rincón-Ortíz J, Moncayo-Lasso A (2019) Advanced oxidation of antihypertensives losartan and valsartan by photo-electro-Fenton at near-neutral pH using natural organic acids and a dimensional stable anode-gas diffusion electrode (DSA-GDE) system under light emission diode (LED) lighting. Environ Sci Pollut Res Int 26(5):4426–4437. 10.1007/s11356-018-2645-3 29971747 10.1007/s11356-018-2645-3

[CR41] Martínez-Pachón D, Botero-Coy AM, Hernández F, López NL, Torres-Palma RA, Moncayo-Lasso A (2022) Elimination of contaminants of emerging concern and their environmental risk in world-real municipal wastewaters by electrochemical advanced oxidation processes. J Environ Chem Eng 10(3):107803. 10.1016/j.jece.2022.107803

[CR42] Martínez-Pachón D, Echeverry-Gallego RA, Serna-Galvis EA, Villarreal JM, Botero-Coy AM, Hernández F, … Moncayo-Lasso A (2021) Treatment of wastewater effluents from Bogotá - Colombia by the photo-electro-Fenton process: Elimination of bacteria and pharmaceutical. The Science of the Total Environment, 772, 144890. 10.1016/j.scitotenv.2020.144890 10.1016/j.scitotenv.2020.144890 33578165

[CR43] Martínez-Sánchez C, Robles I, Godínez LA (2022) Review of recent developments in electrochemical advanced oxidation processes: application to remove dyes, pharmaceuticals, and pesticides. Int J Environ Sci Technol. 10.1007/s13762-021-03762-9

[CR44] Moreno-Andrés J, Tierno-Galán M, Romero-Martínez L, Acevedo-Merino A, Nebot E (2023) Inactivation of the waterborne marine pathogen Vibrio alginolyticus by photo-chemical processes driven by UV-A, UV-B, or UV-C LED combined with H2O2 or HSO5. Water Res 232:119686. 10.1016/j.watres.2023.119686 36764105 10.1016/j.watres.2023.119686

[CR45] Murrieta MF, Brillas E, Nava JL, Sirés I (2023) Solar photoelectro-Fenton-like process with anodically-generated HClO in a flow reactor: Norfloxacin as a pollutant with a particular structure. Sep Purif Technol 308:122893. 10.1016/j.seppur.2022.122893

[CR46] Nguyen-Sy T, To Q-D, Vu M-N, Nguyen T-D, Nguyen T-T (2021) Predicting the electrical conductivity of brine-saturated rocks using machine learning methods. J Appl Geophys 184:104238. 10.1016/j.jappgeo.2020.104238

[CR47] Nikaido H, Nikaido K, Harayama S (1991) Identification and characterization of porins in Pseudomonas aeruginosa. J Biol Chem 266(2):770–779. 10.1016/S0021-9258(17)35239-0 1702438

[CR48] Numberger D, Ganzert L, Zoccarato L, Mühldorfer K, Sauer S, Grossart H-P, Greenwood AD (2019) Characterization of bacterial communities in wastewater with enhanced taxonomic resolution by full-length 16S rRNA sequencing. Sci Rep 9(1):9673. 10.1038/s41598-019-46015-z 31273307 10.1038/s41598-019-46015-z PMC6609626

[CR49] Ohtomo R, Saito M (2001) Increase in the Culturable Cell Number of Escherichia coli during Recovery from Saline Stress: Possible Implication for Resuscitation from the VBNC State. Microb Ecol 42(2):208–214. 10.1007/s002480000103 12024284 10.1007/s002480000103

[CR50] Önnby L, Salhi E, McKay G, Rosario-Ortiz FL, von Gunten U (2018) Ozone and chlorine reactions with dissolved organic matter - Assessment of oxidant-reactive moieties by optical measurements and the electron donating capacities. Water Res 144:64–75. 10.1016/j.watres.2018.06.059 30014980 10.1016/j.watres.2018.06.059

[CR51] Pai C-W, Wang G-S (2022) Treatment of PPCPs and disinfection by-product formation in drinking water through advanced oxidation processes: Comparison of UV, UV/Chlorine, and UV/H2O2. Chemosphere 287(Pt 3):132171. 10.1016/j.chemosphere.2021.132171 34537457 10.1016/j.chemosphere.2021.132171

[CR52] Palma-Goyes RE, Sosa-Rodríguez FS, Rivera FF, Vazquez-Arenas J (2022) Modeling the sulfamethoxazole degradation by active chlorine in a flow electrochemical reactor. Environ Sci Pollut Res Int 29(28):42201–42214. 10.1007/s11356-021-16154-w 34467494 10.1007/s11356-021-16154-w

[CR53] Pitarch E, Medina C, Portolés T, López FJ, Hernández F (2007) Determination of priority organic micro-pollutants in water by gas chromatography coupled to triple quadrupole mass spectrometry. Anal Chim Acta 583(2):246–258. 10.1016/j.aca.2006.10.012 17386553 10.1016/j.aca.2006.10.012

[CR54] Popova S, Tsenter I, Garkusheva N, Beck SE, Matafonova G, Batoev V (2021) Evaluating (sono)-photo-Fenton-like processes with high-frequency ultrasound and UVA LEDs for degradation of organic micropollutants and inactivation of bacteria separately and simultaneously. J Environ Chem Eng 9(3):105249. 10.1016/j.jece.2021.105249

[CR55] Prasad A, Gänzle M, Roopesh MS (2019) Inactivation of escherichia coli and salmonella using 365 and 395 nm high intensity pulsed light emitting diodes. Foods 8(12):679. 10.3390/foods8120679 31847186 10.3390/foods8120679 PMC6963940

[CR56] Rehman F, Sayed M, Khan JA, Shah NS, Khan HM, Dionysiou DD (2018) Oxidative removal of brilliant green by UV/S2O82-, UV/HSO5- and UV/H2O2 processes in aqueous media: A comparative study. J Hazard Mater 357:506–514. 10.1016/j.jhazmat.2018.06.012 30008383 10.1016/j.jhazmat.2018.06.012

[CR57] Rodríguez FA, Mateo MN, Aceves JM, Rivero EP, González I (2013) Electrochemical oxidation of bio-refractory dye in a simulated textile industry effluent using DSA electrodes in a filter-press type FM01-LC reactor. Environ Technol 34(5–8):573–583. 10.1080/09593330.2012.706645 23837306 10.1080/09593330.2012.706645

[CR58] Şahinkaya S (2013) COD and color removal from synthetic textile wastewater by ultrasound assisted electro-Fenton oxidation process. J Ind Eng Chem 19(2):601–605. 10.1016/j.jiec.2012.09.023

[CR59] Schymanski EL, Jeon J, Gulde R, Fenner K, Ruff M, Singer HP, Hollender J (2014) Identifying small molecules via high resolution mass spectrometry: Communicating confidence. Environ Sci Technol 48(4):2097–2098. 10.1021/es5002105 24476540 10.1021/es5002105

[CR60] Scialdone O, Proietto F, Galia A (2021) Electrochemical production and use of chlorinated oxidants for the treatment of wastewater contaminated by organic pollutants and disinfection. Curr Opin Electrochem 27:100682. 10.1016/j.coelec.2020.100682

[CR61] Serna-Galvis EA, Salazar-Ospina L, Jiménez JN, Pino NJ, Torres-Palma RA (2020) Elimination of carbapenem resistant Klebsiella pneumoniae in water by UV-C, UV-C/persulfate and UV-C/H2O2. Evaluation of response to antibiotic, residual effect of the processes and removal of resistance gene. J Environ Chem Eng 8(1):102196. 10.1016/j.jece.2018.02.004

[CR62] Seymour I, O’Sullivan B, Lovera P, Rohan JF, O’Riordan A (2020) Electrochemical detection of free-chlorine in Water samples facilitated by in-situ pH control using interdigitated microelectrodes. Sens Actuators, B Chem 325:128774. 10.1016/j.snb.2020.128774

[CR63] Shi Y, Chen J, Xiao S, Zhang Y, Zhou X (2023) Revisiting the Mineralization of Organic Contaminants in Advanced Oxidation Processes. ACS ES&T Water 3(11):3449–3451. 10.1021/acsestwater.3c00523

[CR64] Song K, Mohseni M, Taghipour F (2019) Mechanisms investigation on bacterial inactivation through combinations of UV wavelengths. Water Res 163:114875. 10.1016/j.watres.2019.114875 31344504 10.1016/j.watres.2019.114875

[CR65] Spuhler D, Andrés Rengifo-Herrera J, Pulgarin C (2010) The effect of Fe2+, Fe3+, H2O2 and the photo-Fenton reagent at near neutral pH on the solar disinfection (SODIS) at low temperatures of water containing Escherichia coli K12. Appl Catal B 96(1–2):126–141. 10.1016/j.apcatb.2010.02.010

[CR66] Stefan MI (Ed.) (2017) Advanced oxidation processes for water treatment: fundamentals and applications*.* IWA publishing

[CR67] Sun H, Wang J, Jiang Y, Shen W, Jia F, Wang S, … Zhang L (2019) Rapid Aerobic Inactivation and Facile Removal of Escherichia coli with Amorphous Zero-Valent Iron Microspheres: Indispensable Roles of Reactive Oxygen Species and Iron Corrosion Products. Environmental Science & Technology, 53(7), 3707–3717. 10.1021/acs.est.8b06499 10.1021/acs.est.8b06499 30817131

[CR68] Tangphant K, Sudaprasert K, Channarong S (2014) Mathematical modeling of electrical conductivity in electrolyte solution between two gas-evolving electrodes. Russ J Electrochem 50(3):253–259. 10.1134/S1023193514030136

[CR69] Tian F-X, Ye W-K, Xu B, Hu X-J, Ma S-X, Lai F, … Wang B (2020) Comparison of UV-induced AOPs (UV/Cl2, UV/NH2Cl, UV/ClO2 and UV/H2O2 ) in the degradation of iopamidol: Kinetics, energy requirements and DBPs-related toxicity in sequential disinfection processes. Chemical Engineering Journal (Lausanne, Switzerland : 1996), 398, 125570. 10.1016/j.cej.2020.125570 10.1016/j.cej.2020.125570 PMC7260538 32508521

[CR70] Wallace RL, Ouellette M, Jean J (2019) Effect of UV-C light or hydrogen peroxide wipes on the inactivation of methicillin-resistant Staphylococcus aureus, Clostridium difficile spores and norovirus surrogate. J Appl Microbiol 127(2):586–597. 10.1111/jam.14308 31077510 10.1111/jam.14308

[CR71] Wan Q, Cao R, Wen G, Xu X, Xia Y, Wu G, … Huang T (2022) Efficacy of UV-LED based advanced disinfection processes in the inactivation of waterborne fungal spores: Kinetics, photoreactivation, mechanism and energy requirements. Sci Total Environ, 803, 150107. 10.1016/j.scitotenv.2021.150107 10.1016/j.scitotenv.2021.150107 34525763

[CR72] Wang C, Moore N, Bircher K, Andrews S, Hofmann R (2019) Full-scale comparison of UV/H2O2 and UV/Cl2 advanced oxidation: The degradation of micropollutant surrogates and the formation of disinfection byproducts. Water Res 161:448–458. 10.1016/j.watres.2019.06.033 31228664 10.1016/j.watres.2019.06.033

[CR73] Wang Y, Xue Y, Zhang C (2020) Generation and application of reactive chlorine species by electrochemical process combined with UV irradiation: Synergistic mechanism for enhanced degradation performance. Sci Total Environ 712:136501. 10.1016/j.scitotenv.2020.136501 31931214 10.1016/j.scitotenv.2020.136501

[CR74] Wang Junshu, Bu L, Wu Y, Sun J, Li G, Zhou S (2022) Disinfection profiles and mechanisms of E. coli, S. aureus, and B. subtilis in UV365/chlorine process: Inactivation, reactivation, and DBP formation. Sep Purif Technol 287:120584. 10.1016/j.seppur.2022.120584

[CR75] Wu J, Cheng S, Duan Y-Z, Shen S-Q, Jiang B-C, Li Y, … Li A-M (2021) Kinetics and efficacy of membrane/DNA damage to Bacillus subtilis and autochthonous bacteria during UV/chlorine treatment under different pH and irradiation wavelengths. Chem Eng J, 422, 129885. 10.1016/j.cej.2021.129885

[CR76] Yao X, Jericho M, Pink D, Beveridge T (1999) Thickness and elasticity of gram-negative murein sacculi measured by atomic force microscopy. J Bacteriol 181(22):6865–6875. 10.1128/JB.181.22.6865-6875.1999 10559150 10.1128/jb.181.22.6865-6875.1999 PMC94159

[CR77] Yin R, Dai T, Avci P, Jorge AES, de Melo WCMA, Vecchio D, … Hamblin MR (2013) Light based anti-infectives: ultraviolet C irradiation, photodynamic therapy, blue light, and beyond. Current Opinion in Pharmacology, 13(5), 731–762. 10.1016/j.coph.2013.08.009 10.1016/j.coph.2013.08.009 PMC3831650 24060701

[CR78] Yoon Y, Jee H, Song SH, Hwang M-H, Chae K-J, Kim B, Yang E (2023) Combination of H2O2-producing microbial desalination cells and UV/H2O2 advanced oxidation process: Water salinity reduction and microbial inactivation. J Environ Chem Eng 11(3):110110. 10.1016/j.jece.2023.110110

[CR79] Zeng F, Cao S, Jin W, Zhou X, Ding W, Tu R, … Ding F (2020) Inactivation of chlorine-resistant bacterial spores in drinking water using UV irradiation, UV/Hydrogen peroxide and UV/Peroxymonosulfate: Efficiency and mechanism. Journal of Cleaner Production, 243, 118666. 10.1016/j.jclepro.2019.118666

