# Supplementary material for: Synergistic effects of a sequential recirculation electrochemical system combined with low-cost UV-LEDs on the gram-negative bacteria inactivation
Source: Environ Sci Pollut Res Int. 2024 Dec 21;32(2):904–24. doi: 10.1007/s11356-024-35297-0 (PMC11732779; doi:10.1007/s11356-024-35297-0)
Supplement: Supplementary file 1 — Supplementary file1 (DOCX 3156 KB) [file 11356_2024_35297_MOESM1_ESM.docx]

**SUPPLEMENTARY MATERIAL**

**Synergistic effects of a sequential recirculation electrochemical system combined with low-cost UV-LEDs on the gram-negative bacteria inactivation**

Paula Andrea Espinosa-Barrera^1,2^, Efraím A. Serna-Galvis^3,4^, Ricardo Antonio Torres-Palma^3^, David Izquierdo-Sandoval^5^, Félix Hernández^5^, Diana Martínez-Pachón^1^, Alejandro Moncayo-Lasso^1*^

*^1^Grupo de Investigación en Ciencias Biológicas y Químicas, Facultad de Ciencias, Universidad Antonio Nariño, Bogotá D.C., Colombia.*

*^2^Doctorado en Ciencia Aplicada - DCA, Universidad Antonio Nariño, Bogotá D.C., Colombia.*

*^3^Grupo de Investigación en Remediación Ambiental y Biocatálisis (GIRAB), Instituto de Química, Facultad de Ciencias Exactas y Naturales, Universidad de Antioquia UdeA, Calle 70 No. 52-21, Medellín, Colombia.*

*^4^Grupo de Catalizadores y Adsorbentes (CATALAD), Instituto de Química, Facultad de Ciencias Exactas y Naturales, Universidad de Antioquia UdeA, Calle 70 No. 52-21, Medellín, Colombia.*

*^5^Environmental and Public Health Analytical Chemistry, Research Institute for Pesticides and Water, University Jaume I, Castellón, Spain.*

**Corresponding author:**

* Alejandro Moncayo-Lasso (e-mail: [amoncayo@uan.edu.co](mailto:amoncayo@uan.edu.co))

**SUPPLEMENTARY TEXTS**

**Text S1. Safety measures to prevent contamination and washing protocol.**

The pre- and post-experiment disinfection protocol was carried out with rigorous biosafety measures. All manipulations, from water inoculation to Petri dish seeding and sample preparation for viability assessment by flow cytometry, were performed inside a laminar flow hood to prevent external contamination. Before each experiment, all autoclavable materials, including the NaCl solution for treatment, water for dilutions, precipitation beakers, borosilicate container used as a radiation tank, connection hoses, pipette tips, wooden swabs for inoculation, 1 mL Eppendorf tubes for Petri dish seeding, and 250 μL tubes for flow cytometry, as well as Petri dishes and nutrient agar, were sterilized using an autoclave (All American 21-1/2 Quart capacity - Model: 921, Sterilization zone: 121°C for 15 minutes).

Furthermore, the entire recirculation system was washed with 70% ethanol before and after each assay, followed by several rinses with ultrapure water to completely remove alcohol. Additionally, before and after assembling the electrochemical flow cell, the components were left inside the laminar flow hood with activated UV radiation from the cabin's UV mercury lamp for disinfection. The work area was also disinfected with 70% ethanol, and whenever possible, UV radiation was applied for further disinfection.

Within the contamination safety measures, the following considerations were implemented: i) Whenever plate counting evaluations were conducted, an uncovered Petri dish was left in the laminar flow hood as a control for ambient contamination. If this dish became contaminated, it indicated that the laminar flow hood was not properly decontaminated. However, no ambient contamination was observed in any of the experiments. ii) Petri dishes containing nutrient agar were stored upside down, either in a refrigerator for initial inoculation intended for future work or in the incubator for sample Petri dishes used in plate counting. This was done to prevent the risk of contamination from airborne particles and water condensation that could affect microbial growth. iii) All solutions (NaCl solution, nutrient agar, and distilled water) were prepared and sterilized on the same day as the experiments were conducted. Whenever possible, materials were not stored after sterilization to ensure maximum sterility and to avoid potential contaminations.

**Text S2. Model of conductivity.**

In the electrolytic process, the electrode surfaces are covered by gas bubbles, and the transfer of ions in the electrolyte is considered as a resistance to charge transport. The physical model of ohmic resistance is based on Ohm's law, as shown in Eq. (S1):

$R=\frac{L}{\sigma_{B}A´}$ (S1)

where *R* is the resistance of the electrolyte, *L* is the distance between electrodes, *А* is the surface area of the electrode. The mixture conductivity (*σ_B_*) is determined from the void fraction. It is decreased due to the presence of the gas in the electrolyte. The conductivity can be calculated using Bruggeman correction as expressed in Eq. (S2):

$\sigma_{B}= \sigma_{0}{(1-\alpha_{total})}^{1.5}$ (S2)

where σ_0_ is the conductivity with no bubbles and αtotal is the total void fraction.

This model was implemented before conducting any experiment, either for monitoring oxidant generation or microorganism elimination. Its purpose was to establish the minimum and maximum conductivity limits, considering the distance between the electrodes and their area. These limits are applicable in any cell configuration, whether for the generation of ACS (Steel - DSA), H_2_O_2_ (GDE - Graphite), or a combination of both (GDE - DSA). This applicability is due to the presence of the double layer (catalytic and diffusion layer) and a defined electrode/electrolyte interface in both types of electrodes, DSA and GDE. It is important to mention that this model was not designed to provide a detailed electrochemical characterization of the cell, so bulk convection was neglected in this calculation.

**Text S3. Identification of chlorinated DBPs.**

For the solid phase extraction, 10 mL of the sample was passed through the SPE cartridge previously conditioned with 6 mL of methanol, 6 mL of ethyl acetate:DCM (50:50), 6 mL of methanol, and 6 mL of deionized water, avoiding dryness. After loading the sample, cartridges were washed with 3 mL of deionized water and dried by passing air under a vacuum for 30 minutes. Afterward, 5 mL of ethyl acetate:DCM (50:50) was used to elute the DBPs from the cartridge. Finally, the resulting extracts were evaporated under a gentle nitrogen stream at 40ºC and redissolved with 50 µL of hexane. A sample of 10 mL of deionized water was subjected to the entire process as a blank.

Data were acquired using a Thermo Scientific™ Q Exactive™ GC hybrid quadrupole-Orbitrap mass spectrometer. Sample injection was performed with a TriPlus RSH autosampler (Thermo Scientific, Bremen, Germany). For GC separation, a Thermo Scientific™ TRACE™ 1310 GC was used. The injector temperature was set at 300°C in split/splitless mode at a flow rate of 50 mL/min, a purge time of 1.0 min, and an injection volume of 1 μL. An HP-5MS capillary column (Thermo Fisher ScientificTM, Palo Alto, CA, USA) of 30 m × 0.25mm i.d. × 0.25µm film thickness and helium at 1 mL/min was used with a temperature program that started at 90 ºC for 1 min, increased to 330 ºC at 5 ºC/min and held for 4 min (total chromatographic analysis time: 53 min). C8-C40 alkane series was used for the external non-isothermal retention index (RI). The electron ionization was performed at 70 eV in positive, with source and transfer temperatures set at 270 °C and 330 °C, respectively. Data were acquired in full scan mode at a rate of 3.7 scan/s over a m/z range of 40 to 750 m/z at 60,000 mass resolution (FWHM at m/z 272). The mass calibration procedure was performed daily (perfluorotributylamine). The instrument was controlled by Xcalibur 4.0 software (Thermo Scientiﬁc, Waltham, MA, USA).

Compound discover (Thermo Scientiﬁc, Waltham, MA, USA, v3.3) was employed for spectral deconvolution, peak integration, and annotation by NIST Library (2024). The initial settings for deconvolution software were S/N greater than 3, mass error of ± 5 ppm, total ion chromatogram (TIC) intensity threshold of 1,000,000, and ion overlap windows of 98%. The compounds were characterized by their Rt and accurate m/z of molecular ion (if present). Missing values were imputed using the smallest peak area detected in a sample divided by 2. Any compounds with a sample-to-blank ratio of less than 5 were set to be ignored. They were tentatively identified by automated comparison of the deconvoluted mass spectra with the ones present in the National Institute of Standards and Technology (NIST) 2024 library (EI spectra of 306,643 compounds and retention index, RI, values of 139,382 compounds) with reversed search index (RSI), high-resolution filtering (HRF) value and a Deviation of the Retention Index (% ΔRI) match higher than 700, 80 and lower than 5 %, respectively. Only annotated compounds containing Cl in the proposed molecular formula were considered. Fig. S6 illustrates the tentative identification of 1,2,3,4-tetrachlorocyclopenta-1,3-diene in the T45 sample. The identification level was assigned according to the five-level classification proposed for environmental applications (Schymanski et al., 2014). Level 2 has been assigned when an exact structure is matched in the NIST library in terms of SI, HRF, and RI criteria. Level 3 has been assigned when different structures match the NIST library and meet the SI, HRF, and RI criteria.

**SUPPLEMENTARY FIGURES**


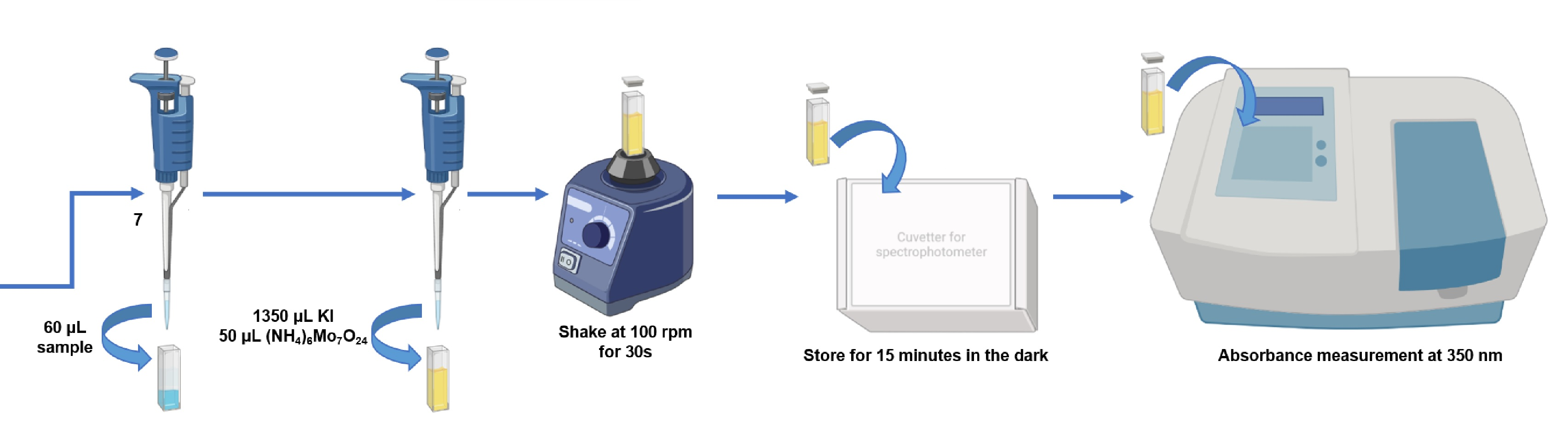
**Figure S1.** Schematic diagram of the method and procedure for measuring oxidants through spectrophotometry. Number 7 represents the liquid samples taken from the electrochemical system of sequential recirculation assisted by UVA-LEDs (UV-LEDs/GDE/DSA) (Fig. 1A). The blue line represents the workflow.


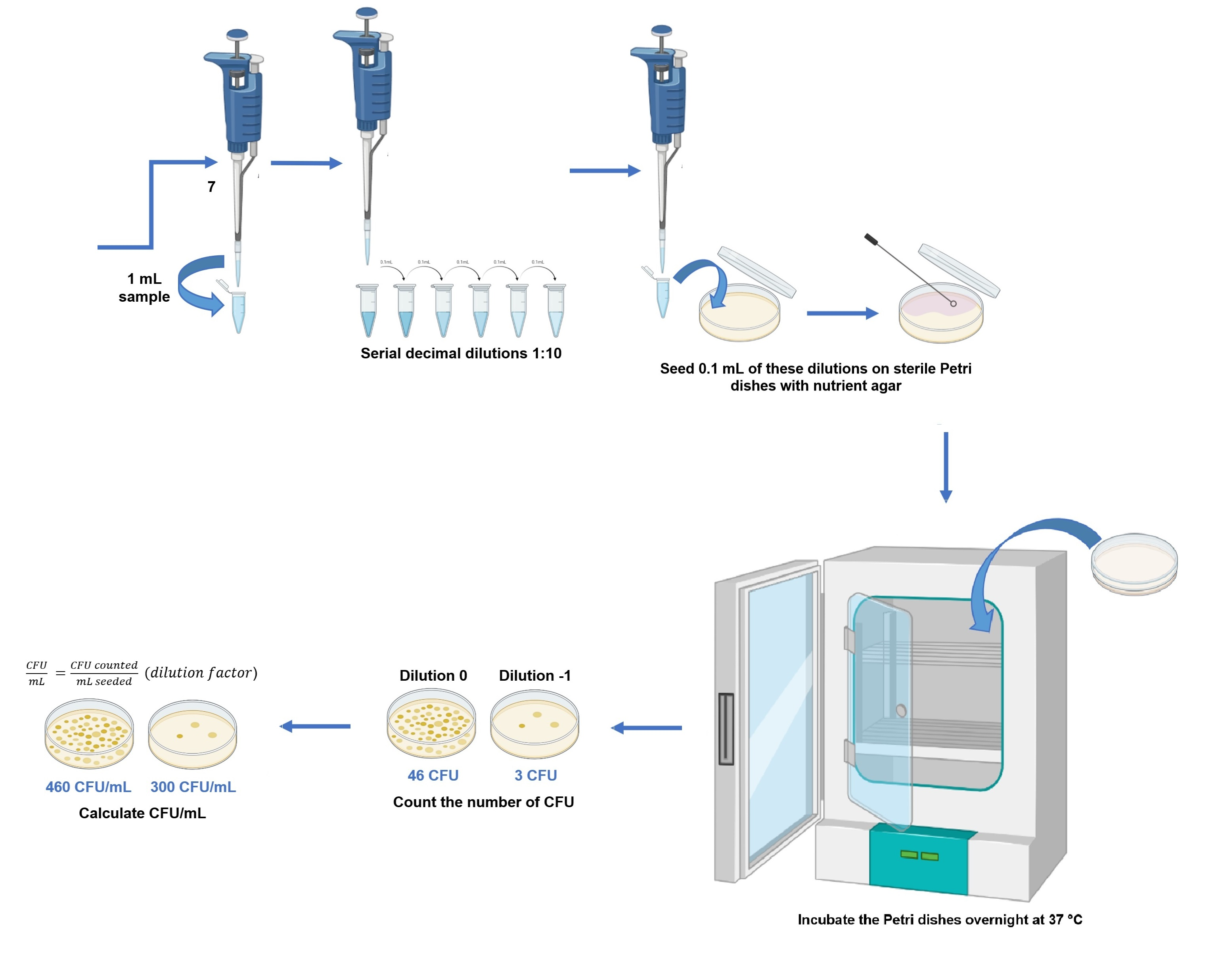
**Figure S2.** Schematic diagram of the method and procedure for the CFU plate count. Number 7 represents the liquid samples taken from the sequential recirculation electrochemical system assisted by UVA-LEDs (UV-LEDs/GDE/DSA) (Fig. 1A). The blue line represents the workflow.


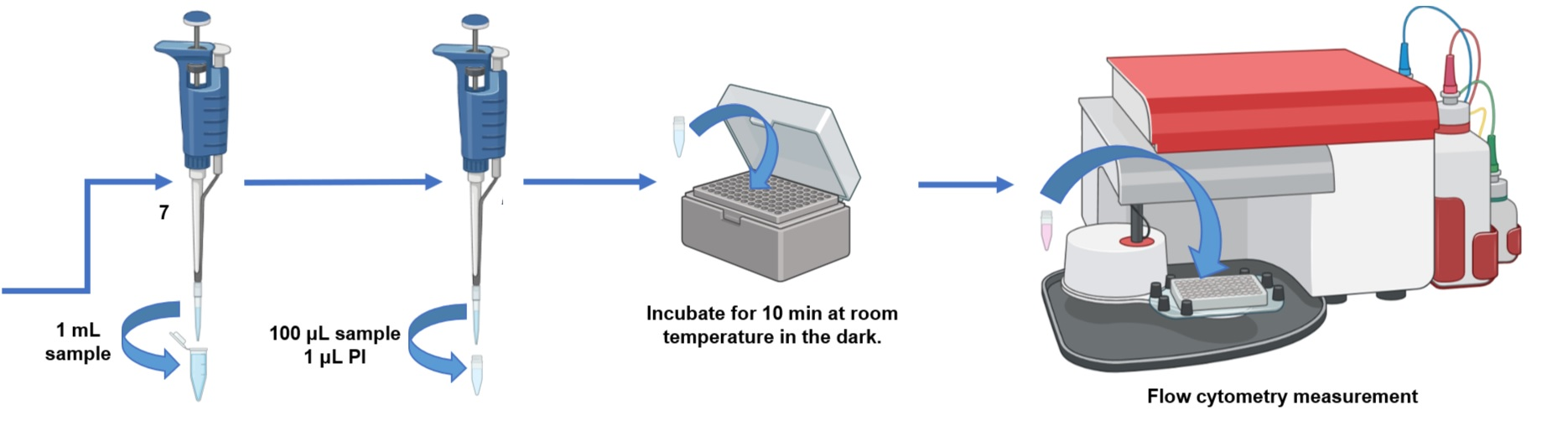
**Figure S3.** Schematic diagram of the method and procedure for the evaluation of bacterial viability by flow cytometry. Number 7 represents the liquid samples taken from the sequential recirculation electrochemical system assisted by UVA-LEDs (UV-LEDs/GDE/DSA) (Fig. 1A). The blue line represents the workflow.

**A**

**B**

**C**

**D**

**Figure S4**. Controls on the generation of oxidants. **A.** Oxidants in the electrochemical system GDE/DSA and DSA with and without bacteria. System conditions: pH_initial_: 6.45, current density: 7.5 mA/cm^2^, flow rate: 241 mL/min, [NaCl]: 0.0125 mol/L, radiation source: N/A, *E. coli* concentration_initial_: 3x10^8^ CFU/mL. **B.** Effect of supporting electrolyte concentration on the generation of oxidants by the GDE/DSA electrochemical system without bacteria. System conditions: pH_initial_: 6.50, current density: 7.5 mA/cm^2^, flow rate: 241 mL/min, radiation source: N/A. **C.** Effect of current density on the generation of oxidants by GDE/DSA electrochemical system without bacteria. System conditions: pH_initial_: 6.49, flow rate: 241 mL/min, [NaCl]: 0.0125 mol/L, radiation source: N/A. **D.** Comparison of oxidant generation by individual subsystems (DSA), binary subsystems (UV-LED/DSA and GDE/DSA), and the coupled system (UV-LED/ GDE/DSA), followed without bacteria. System conditions: Average pH_initial_: 6.50, Average pH_final_: 6.79, current density: 7.5 mA/cm^2^, flow: 241 mL/min, [NaCl]: 0.0125 mol/L, radiation source: UV-LEDs. Bars represent the average oxidant generation **(A, B, C, and D)** across all treatments with a p ≤ 0.05 (n=9, with three replicates of the experiment and three replicates of measurement).

**Figure S5.** States in the evolution of flow cytometry results and PI staining during the treatment of *E. coli* (Right - orange color) and *P. aeruginosa* (Left - mint green color) using the UV-LEDs/GDE/DSA system. System conditions: Average pH_initial_: 6.59, average pH_final_: 6.74, current density: 7.5 mA/cm^2^, flow: 241 mL/min, [NaCl]: 0.0125 mol/L, radiation source: UV-LEDs, *E. coli* concentration_initial_: 3x10^8^ CFU/mL. Conditions for evaluation: Volume of solution: 100 µL, Temperature: 25.3 °C. Average of three replicated experiments and three replicated measurements, p ≤ 0.05 (n=9).

**Figure S6.** Evolution of cell unviability during treatment of *E. coli* by individual subsystems (UV-LEDs, GDE, and DSA), binary subsystems (UV-LEDs/GDE, UV-LEDs/DSA, and GDE/DSA), and the coupled system (UV-LEDs/GDE/DSA), followed by flow cytometry. System conditions: Average pH_initial_: 6.50, average pH_final_: 6.79, current density: 7.5 mA/cm^2^, flow: 241 mL/min, [NaCl]: 0.0125 mol/L, radiation source: UV-LEDs, *E. coli* concentration_initial_: 3x10^8^ CFU/mL. Conditions for evaluation: Volume of solution: 100 µL, Temperature: 25.3 °C. Average of three replicated experiments and three replicated measurements, p ≤ 0.05 (n=9).


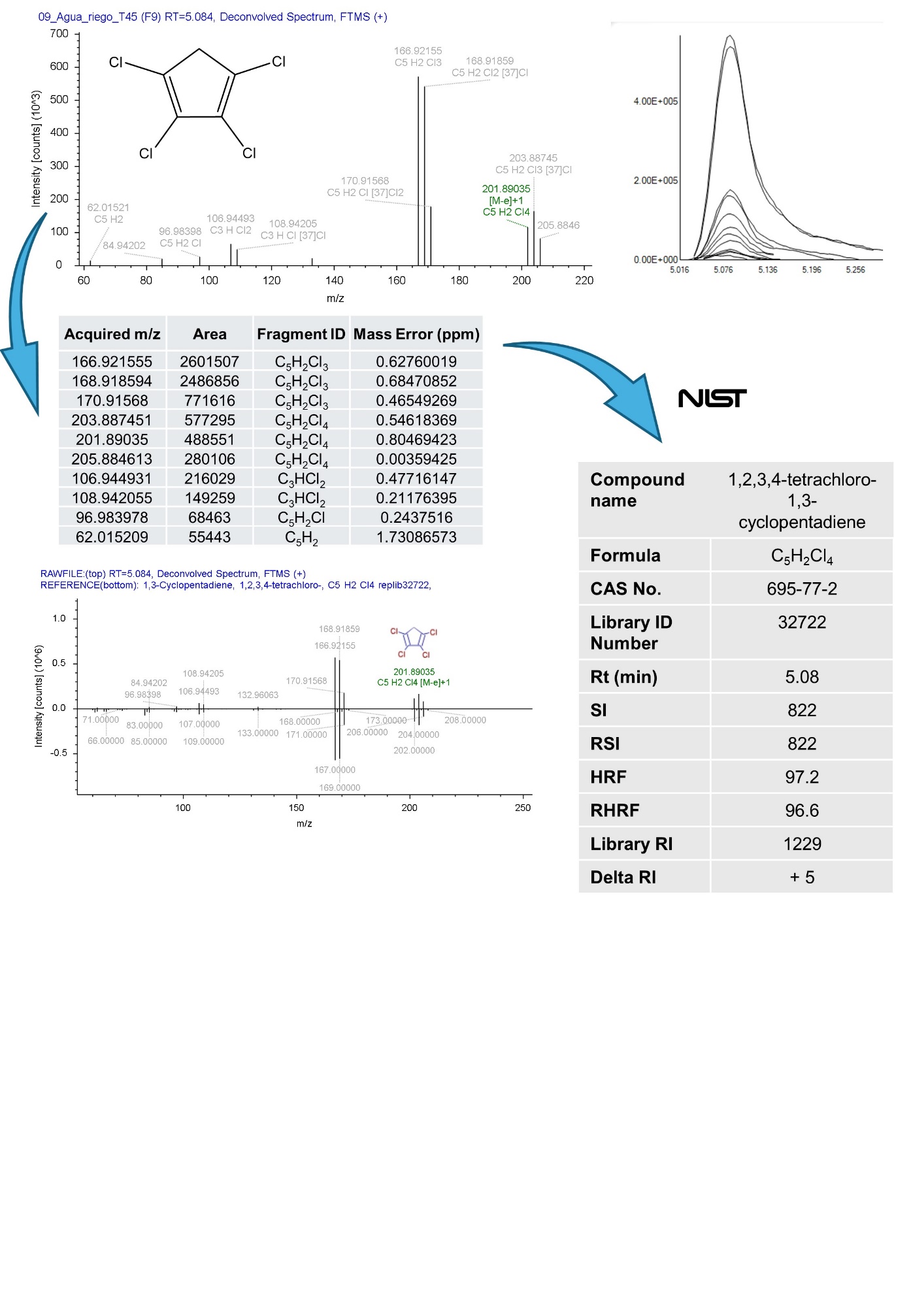


**Figure S7**. Example of 1,2,3,4-tetrachlorocyclopenta-1,3-diene (CAS no. 695-77-2) tentative identification. Relevant parameters: retention time (Rt), search index (SI), reversed SI (RSI), high-resolution filtering (HRF) value, reversed high-resolution filtering (RHRF), and linear retention index (LRI).

**SUPPLEMENTARY TABLES**

**Table S1.** Operation configuration and parameters of treated and untreated solutions for different electrochemical systems and subsystems in recirculation.

| **System or subsystem** | **Electrochemical cell**  **(*ON/OFF*)^a^** | | **Radiation UV-LEDs**  **(*ON/ OFF*)^b^** | **Aeration**  **(*ON/OFF*)^c^** | **Dissolved oxygen**  **(mg O_2_/L)^d^** | | **pH^d^** | | **Temperature (°C)^d^** |
| --- | --- | --- | --- | --- | --- | --- | --- | --- | --- |
|  | **Cathode** | **Anode** |  |  | **Initial** | **Final^e^** | **Initial** | **Final^e^** |  |
| UV-LEDs/GDE/DSA | ***ON*** | | ***ON*** | ***ON*** | 8.52 ^f^ | 8.21 | 6.59 | 6.74 | 25.2 |
|  | GDE | DSA |  |  |  |  |  |  |  |
| GDE/DSA | ***ON*** | | ***OFF*** | ***ON*** | 8.44 ^f^ | 8.18 | 6.52 | 6.61 | 25.4 |
|  | GDE | DSA |  |  |  |  |  |  |  |
| UV-LEDs/DSA | ***ON*** | | ***ON*** | ***OFF*** | 6.84 | 6.71 | 6.45 | 6.93 | 25.6 |
|  | Steel | DSA |  |  |  |  |  |  |  |
| UV-LEDs/GDE | ***ON*** | | ***ON*** | ***ON*** | 8.43 ^f^ | 8.07 | 6.57 | 6.92 | 25.0 |
|  | GDE | Graphite |  |  |  |  |  |  |  |
| DSA | ***ON*** | | ***OFF*** | ***OFF*** | 6.56 | 6.42 | 6.69 | 7.17 | 25.6 |
|  | Steel | DSA |  |  |  |  |  |  |  |
| GDE | ***ON*** | | ***OFF*** | ***ON*** | 8.49 ^f^ | 8.13 | 6.32 | 6.76 | 25.7 |
|  | GDE | Graphite |  |  |  |  |  |  |  |
| UV-LEDs | ***OFF*** | | ***ON*** | ***OFF*** | 6.97 | 6.84 | 6.41 | 6.45 | 25.1 |
|  | GDE | DSA |  |  |  |  |  |  |  |

^a^ ***ON***: It implies the generation of oxidants.

^b^ ***ON***: Implies constant radiation of the solution in the radiation tank (Fig. 1A).

^c^ ***ON***: It implies the saturation with O_2_ of the solution from 15 minutes before the treatments.

^d^ Average of three replicated experiments and three replicated measurements, p ≤ 0.05 (n=9).

^e^ Final treatment time at 10 minutes.

^f^ Measurement taken after 15 minutes with O_2_ saturation of the solution required before starting the treatment.
